# An Integrated Testing Strategy for Ecotoxicity (ITS‐ECO) Assessment in the Marine Environmental Compartment using *Mytilus* spp.: A Case Study using Pristine and Coated CuO and TiO_2_ Nanomaterials

**DOI:** 10.1002/etc.5313

**Published:** 2022-04-11

**Authors:** Mona Connolly, Simon Little, Mark G. J. Hartl, Teresa F. Fernandes

**Affiliations:** ^1^ Institute of Life and Earth Sciences Heriot‐Watt University Edinburgh United Kingdom

**Keywords:** Titanium dioxide and copper(II) oxide nanomaterials, Functionalization, Integrated testing strategies, Integrated approach to testing and assessment, Bioaccumulation, *Mytilus* spp., Marine, Antioxidant defences

## Abstract

An integrated testing strategy for ecotoxicity assessment (ITS‐ECO) was developed to aid in the hazard and fate assessment of engineered nanomaterials (ENMs) deposited in marine environments using the bivalve *Mytilus* spp. as a test species. The ENMs copper(II) oxide (CuO) and titanium dioxide (TiO_2_), either in pristine form (core) or with functionalized coatings (polyethylene glycol [PEG], carboxyl [COOH], and ammonia [NH_3_]) were selected as case study materials based on their production levels and use. High‐throughput in vitro testing in Tier 1 of the ITS‐ECO revealed CuO ENMs to elicit cytotoxic effects on lysosomes of hemocytes of mussels, with the hazard potential CuO PEG > CuO COOH > CuO NH_3_ > CuO core, whereas TiO_2_ ENMs were not cytotoxic. Genotoxicity in hemocytes as well as gill cells of mussels following in vivo exposure (48 h) to CuO ENMs was also seen. Longer in vivo exposures in Tier 2 (48 h–21 days) revealed subacute and chronic oxidative effects for both CuO and TiO_2_ ENMs, in some cases leading to lipid peroxidation (core TiO_2_ ENMs). In Tier 3 bioaccumulation studies, distinct patterns of uptake for Cu (predominantly in gills) and Ti (predominantly in digestive glands) and between the different core and coated ENMs were found. Clear NM‐specific and coating‐dependent effects on hazard and fate were seen. Overall, using a tiered testing approach, the ITS‐ECO was able to differentiate the hazard (acute, subacute, and chronic effects) posed by ENMs of different compositions and coatings and to provide information on fate for environmental risk assessment of these ENMs. *Environ Toxicol Chem* 2022;41:1390–1406. © 2022 The Authors. *Environmental Toxicology and Chemistry* published by Wiley Periodicals LLC on behalf of SETAC.

## Graphical abstract

Framework for ecotoxicity testing of engineered nanomaterials (ENMs) using a marine compartment‐specific Integrated testing strategy (ITS‐ECO) and the marine bivalve mussel *Mytilus* spp.

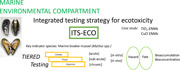

## INTRODUCTION

Marine ecosystems have historically been subject to frequent and extensive anthropogenic pollution, and, according to recent environmental fate models, they are predicted to be major recipients of engineered nanomaterials (ENMs) throughout ENM‐enabled product life cycles (Garner et al., 2017). Integrated testing strategies (ITS), have been proposed as promising tools for satisfying regulatory information requirements for safety assessment (Rovida et al., 2015) while incorporating the Replace, Reduce, and Refine (3R) principles. An ITS also forms a key element of integrated approaches to testing and assessment (IATAs), which moves the focus away from stand‐alone testing using standardized protocols and species to incorporating a range of novel methodologies and tests with considerations for most of the relevant/sensitive nonvertebrate species and mechanisms underlying toxicity.

In the present study we developed an ecotoxicological ITS (ITS‐ECO) focusing on the marine compartment, using the blue mussel, *Mytilus* spp., as a test species. Standardized bivalve tests, for example, those from the International Standards Organization (2015) and ASTM International (2020), are not widely used, despite their benefits in ecotoxicological testing and sensitivity to pollutants of different life stages (e.g., embryo, larval, juveniles; Kennedy et al., 2017), However, their use in nano‐ecotoxicological studies is increasing in significance (2213 Web of Science hits for “nano” and “mussels” in 2021 compared with 533 in 2015; Selck et al., 2016). The use of bivalves specifically in bioaccumulation assessment for ENMs has also recently been highlighted (Kuehr et al., 2021). Specifically, the use of mussel bivalves in an ITS incorporating bioaccumulation assessment may also facilitate decisions on the need for vertebrate testing with fish in a tiered approach, refining testing, and thus incorporating the 3R principles into the developed ITS.

**Figure 1:**
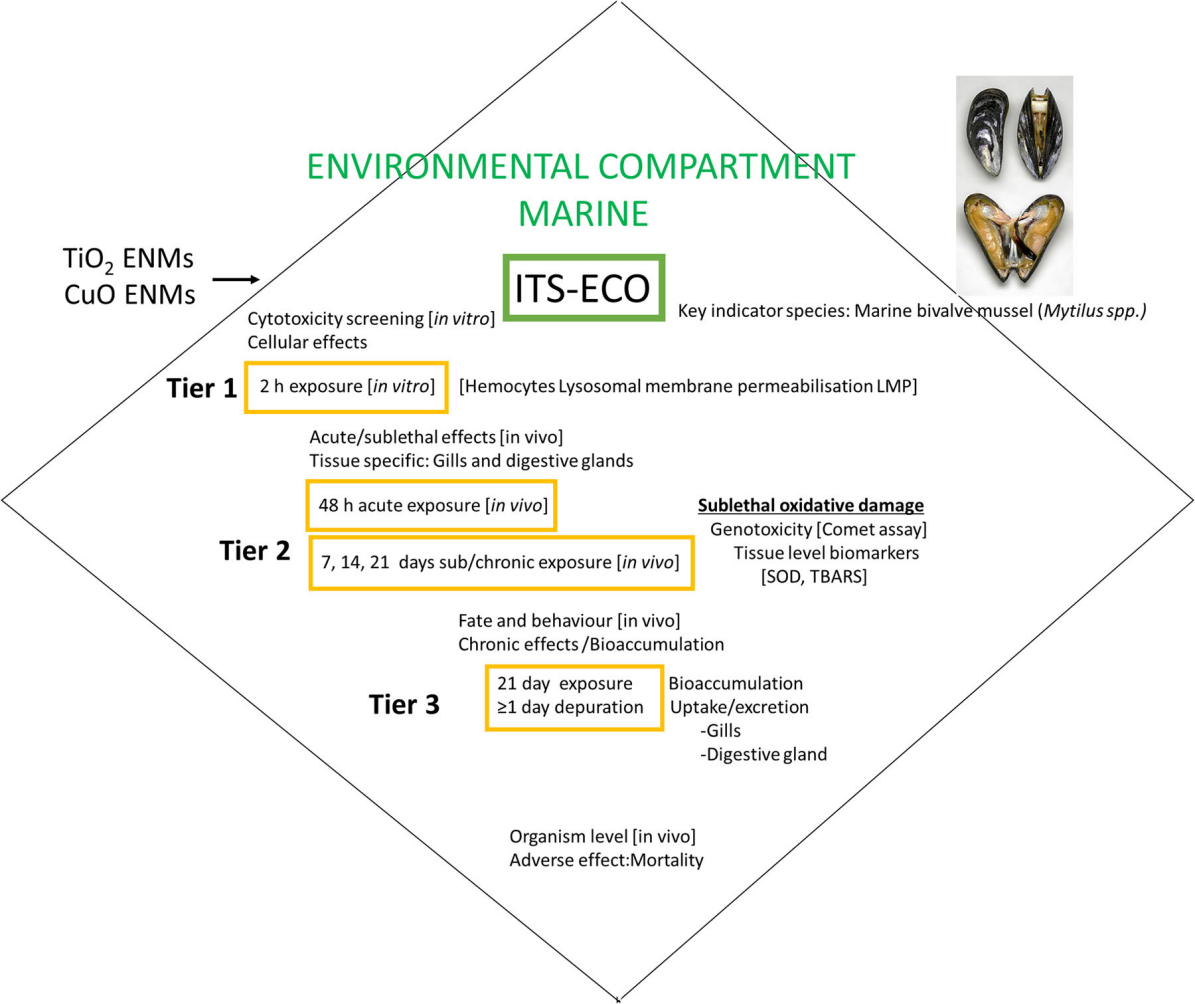
Integrated testing strategy for ecotoxicity (ITS‐ECO) for the marine environment encompassing a tiered approach at the cellular, tissue, and organism levels, including in vitro cellular screening, acute, subacute, and chronic in vivo testing, and engineered nanomaterial (ENM) fate assessment. SOD = superoxide dismutase; TBARS = thiobarbituric acid reactive substance.

In the present study, existing protocols and available literature were used to inform the development of an ITS comprising tiers of increasing complexity (Figure 1). The novelty of this framework lies in the integration of testing at different tiers, systematically, including multiple endpoints and the incorporation of a key invertebrate species in an ITS of an IATA for ecotoxicity testing of ENMs for marine environments.

Two ENMs (copper(II) oxide [CuO] and titanium dioxide [TiO_2_]) were used as case studies and were selected based on their relevance to the marine environment, high production levels, use in marine antifouling paints and coatings (Miller et al., 2020; Szeto et al., 2021), as well as the use of TiO_2_ ENMs in sunscreens (Botta et al., 2011), and the use of CuO ENMs as fertilizers, with the potential for run‐off (Xiong et al., 2017). The ENMs are often functionalized for improved performance, which will also confer a new identity on the material, potentially leading to distinct environmental fate, behavior, and biological effects compared with the core ENM (Perreault et al., 2014). For that reason, and because of the ability of mussels to sort particulates (Al‐Shaeri et al., 2013), we used CuO and TiO_2_ ENMs with a series of functionalizations (carboxyl [COOH], polyethylene glycol [PEG], and ammonia [NH_3_]) to investigate whether distinct hazards and fate are dictated and/or can be controlled by functionalities.

The initial tier of the ITS focuses on high‐throughput cellular cytotoxicity screening using hemocytes (immune cells) from the circulatory fluid (hemolymph) of mussels. Lysosomes are an important organelle in intracellular ENM storage and transport following uptake (the endolysosomal pathway; Stern et al., 2012). Biopersistent/biodurable ENMs can induce lysosomal membrane permeabilization (LMP), which has been highlighted as a means of predicting potential long‐term effects within safety assessments (Organisation for Economic Co‐operation and Development [OECD], 2020). As an initiating step within the oxidative stress adverse outcome pathway, LMP is commonly associated with ENMs, which can lead to a loss of mitochondrial membrane potential, imbalance of reactive oxygen species (ROS), programmed or unprogrammed cellular death (e.g., apoptosis, necrosis, autophagy), and ultimately tissue injury (Gerloff et al., 2017). Consequently, LMP (using the neutral red uptake assay) was prioritized as an high‐throughput screening method, with the aim of linking cellular effects to tissue‐ and organism‐level effects in higher tiers.

Tier 2 of the ITS‐ECO concentrates on sublethal effects in the main target organ tissues (gills and digestive gland) following acute, in vivo exposure. Levels of superoxide dismutase (SOD) activity and the thiobarbituric acid reactive substance (TBARS) assay for lipid peroxidation (LPO) were used in an integrated biomarkers of response approach for oxidative stress. Superoxide dismutase is a first line of defence against oxygen radical (e.g., superoxide anion $O_{2}{•}^{-}$) insult in mussels following metal‐induced oxidative stress (Vlahogianni et al., 2007). Together with monitoring of late oxidative stress effects, that is, LPO via the TBARS assay, an integrated assessment of oxidative stress and cells anti‐oxidative capacity can be monitored.

In Tier 3, we used long‐term, chronic, fate‐driven studies assessing ENM bioaccumulation in key bivalve tissues (gills and digestive gland) and any associated adverse effects. Invertebrate bivalves are alternative test organisms for bioaccumulation assessment, to reduce the need for further testing in vertebrates, in tiered screening tests (Handy et al., 2018). Furthermore, mussels exhibit extraordinary pre‐ and postingestive selective and differential particle‐processing capabilities (capture, rejection, ingestion, absorption, and egestion; Riisgård et al., 2011). Particle selection/capture efficiency is influenced by particle diameter, shape, surface charge, surface chemistry (protein coating), and wettability (hydrophobicity) (Al‐Shaeri et al., 2013; Rosa et al., 2017; Wang et al., 2021). Given the potential for surface functionalization to influence the aforementioned properties, we hypothesize that distinct uptake and accumulation profiles could be evidenced in mussels exposed to core and functionalized ENMs.

## MATERIALS AND METHODS

### ENMs and dispersions

The ENMs were provided by PlasmaChem, as part of the Nanosolutions (2020) European Union project and supplied as dry powders. The CuO and TiO_2_ pristine core ENMs, either uncoated or coated, were as follows: PEG (neutral surface charge), COOH (negative surface charge), or amine (NH_3_; positive surface charge). The TiO_2_ and CuO coated and uncoated ENMs had manufacturer‐reported sizes of 10–20 nm. These materials were characterized within the project, and their physicochemical properties have been presented in various associated publications (CuO: Gajda‐Meissner et al., 2020; Ilves et al., 2019; Kubo et al., 2020; Tatsi et al., 2018; TiO_2_: Vassallo et al., 2018). All relevant information is presented in the Supporting Information, Table S1. Stock suspensions of CuO ENMs and TiO_2_ ENMs were prepared in deionized water (2 mg/ml). Bath sonication (Kerry PUL325 device) was used to aid dispersion (13 W of acoustic power was applied to a sample of 10 ml for 13.6 min; Nanosolutions project standard operating procedure dispersion protocol). Thereafter, for Tier 1 in vitro cytotoxicity testing, working suspensions were prepared at nominal concentrations of 200 µg nanoparticles/ml (1:10 dilution of stock) in Hanks' balanced salt solution (HBSS) osmotically adjusted to 990 mOsm/L using 22.2 g/L NaCl (HBSS+) just prior to exposure. Further 1 in 2 serial dilutions of these working suspensions were made to generate concentration ranges for in vitro testing (3.125–200 µg ENMs/ml). For acute and chronic in vivo exposures, quantities of 24 µl of CuO ENM stock suspensions were diluted with seawater directly into the exposure buckets (2.4 L) to generate exposure concentrations of 20 µg CuO ENM/L for each respective core and coated CuO ENM. This concentration was chosen taking into consideration the relatively low expected environmental concentrations (Zhao et al., 2021), limits of detection and quantification, and also concentrations at which sublethal effects were evidenced (Gomes et al., 2013). Similarly, 360 µl of TiO_2_ ENM stock suspensions were diluted in 2.4 L, generating exposure concentrations of 300 µg TiO_2_ ENM/L. This higher concentration was selected considering the higher TiO_2_ ENM production (Robichaud et al., 2009), predicted environmental concentrations (Zhao et al., 2021), and lack of effects seen in mussels (D'Agata et al., 2014), but also potential sublethal effects evidenced (Banni et al., 2016). Aeration was provided using air stones to maintain dispersion of ENM suspensions. Stock suspensions prepared in deionized water (2 mg/ml) and working suspensions prepared in seawater and HBSS+ (at 200 µg ENM/ml) were characterized using dynamic light scattering (DLS; Malvern Zetasizer, Nano).

### Mussel collection and husbandry

Adult mussels (*Mytilus* spp*.)*, approximately 4–6 cm in shell length were collected from an intertidal location in Musselburgh, 5 miles east of Edinburgh (Scotland, UK; 55.94°N, 3.05°W). Mussels were washed, transferred to 45‐L plastic containers approximately half filled with natural, filtered, and aerated seawater (32–34 PSU salinity, 16 °C), and allowed to acclimatize for at least 1 week under a 12:12‐h light:dark cycle before experimentation. Mussels were fed microalga (40 × 10^6^ cells/L) 24 h after they were transferred to plastic containers (day 2 of acclimatization).

### Hemocyte screening assays

Haemolymph (10 ml) was extracted and pooled from the posterior adductor muscle of 25 healthy mussels using a 21‐gauge hypodermic needle fitted to a 1‐ml syringe. Total cell counts were performed using direct hemocytometer counting, confirming 6–7.5 × 10^5^ cells/ml. Cell viability was assessed using the trypan blue exclusion assay. Cell suspensions with 90% or more viability were selected for use in experiments. A volume of 50 µl cell suspension (cell density 5 × 10^5^ cells/ml) was aliquoted into wells of a 96‐well microplate. Cells were allowed to adhere for 45 min at 16 °C. Excess hemolymph was then removed, and only cells that had adhered to the plate were used. Cells were exposed to 100 µl of concentration ranges of ENMs (3.125–200 µg NM/ml) in triplicate prepared in HBSS,osmotically adjusted to 990 mOsm L using 22.2 g/L NaCl (HBSS+) to match the osmolarity of the mussel hemolymph. A Cu (copper(II) sulfate [CuSO_4_]; Sigma Aldrich) was used as a positive control and served to compare effects between particulate and dissolved material. Following 2 h of exposure, lysosomal membrane permeabilization/destabilization was measured using the neutral red uptake assay according to Babich and Borenfreund (1990). Exposure suspensions were removed, and cells were washed with HBSS+ and incubated with neutral red solution (0.033 mg/ml), prepared in HBSS+, in the dark. After 2 h, to allow dye incorporation, the neutral red solution was removed, the cells were rinsed twice with 200 µl of HBSS+, and the neutral red retained in the cells was extracted with an acidified (1% glacial acetic acid) 50% ethanol/49% Milli‐Q water solution (100 µl/well). Neutral red fluorescence was measured at 532 nm/680 nm (excitation/emission) in a microplate reader (Spectra Max M5). The fluorescent values were corrected for the cell‐free control, normalized against the untreated control cells, and represented as % lysosomal membrane stability compared with untreated control. The median effect concentration (EC50) values were calculated from nonlinear regression–fitted, four‐parametric dose–response curves using GraphPad Prism 5, and exposure concentrations were represented in µg Cu/ml to allow direct comparisons between the core and coated ENMs.

Potential interference from ENM preparations with the assay system (e.g., neutral red dye absorption, fluorescence quenching) was assessed using cell‐free wells treated with the exposure concentration ranges. Following incubation of the ENM exposure suspensions with the neutral red dye (0.33 mg/ml) for 2 h, wells were observed under an optical microscope for any visible dye absorption to ENM aggregates. Any fluorescence quenching was then quantified by adding 100 µl of the acidified ethanol solution used in the assay to the wells containing ENMs and neutral red dye, and the fluorescence was quantified at 532 nm/680 nm (excitation/emission).

### Acute exposures

Experiments were carried out in 5‐L acid‐washed (2% v/v HNO_3_) plastic buckets. Six mussels were used per treatment group in 2.4 L of natural filtered seawater as used under holding conditions. Stock suspension dispersions of NMs prepared in deionized water (2 mg/ml; see details in the previous section, *ENMs and dispersions*) were used to spike treatment groups with NMs and to achieve final nominal concentrations of 20 µg/L (CuO ENMs) and 300 µg/L (TiO_2_ ENMs). Mussels were exposed for 48 h with no food source. Gill and digestive gland tissues were dissected after NM exposure. Tissues were blotted dry, weighed, frozen using liquid nitrogen, and immediately stored at −80 °C prior to biochemical analyses. In addition, haemolymph was extracted for hemocyte genotoxicity analysis (using the comet assay as detailed in the following section of the same name).

### Subchronic and chronic exposures

The same experimental setup of groups of 6 mussels, held in 5‐L plastic buckets (acid‐washed using 2% v/v HNO_3_ prior to use) was used for sub‐/chronic studies. Each group of mussels was exposed in 2.4 L natural, filtered seawater (32–34 PSU salinity) either with no treatment (control) or spiked with a core and coated CuO ENM (20 µg/L) or TiO_2_ ENMs (300 µg/L). Independent experiments were performed for 7‐, 14‐, and 21‐day exposures to ensure that the same number of mussels to exposure volume ratio (6 mussels: 2.4 L) was maintained. Following each 24‐h exposure period, mussels were transferred to clean buckets, which were filled with fresh, natural seawater and spiked with the appropriate ENM as just described. In the 14‐ and 21‐day experiments on days 8 and 15, each group was supplied with a low concentration of microalgae (40 × 10^6^ cells/L) as a food source. Feeding was performed approximately 22 h after the previous medium change to avoid interference with the ENM exposure. Mussels were then allowed to feed for 2 h before being transferred to fresh natural seawater. Gills and digestive glands of 3 mussels/group were removed at the end of the 7, 14, and 21 days of exposure experiments, weighed, and immediately snap‐frozen in liquid nitrogen, and stored at −80 °C. A depuration phase was also included at the end of the 21‐day exposure. Three mussels were allowed to depurate for 24 h in fresh natural seawater containing no ENMs. After this depuration period, gills and digestive glands of depurated mussels were removed, weighed, and stored as just described.

### Biochemical analysis

#### Tissue preparation

Thawed gill and digestive gland tissues were homogenized in 1:5 w/v ice cold buffer (10 mM Tris‐HCl; 0.5 sucrose; 1 mM EDTA, pH 7.6) using a Teflon pestle. Homogenates were then centrifuged at 500 *g* for 15 min (4 °C), and the resultant supernatant was centrifuged again at 12,000 *g* for 30 min (4 °C) before use in biochemical analyses.

#### Protein quantification

Protein concentrations in homogenates were determined using a fluorescamine‐based assay (Udenfriend et al., 1972). Briefly, analysis was performed in duplicate by measuring fluorescence at wavelengths of 390 nm excitation and 475 nm emission in a microplate reader (Spectra Max M5) using bovine serum albumin (Sigma‐Adrich) (0.015–2 mg/ml) as a standard.

#### SOD activity

The SOD activity levels in gill and digestive gland homogenates were determined using an SOD Assay Kit‐WST 19160 (Sigma‐Aldrich). A standard curve was prepared using SOD from bovine erythrocytes (0.2–50 U/mg) to quantitatively assess the enzyme activity from the inhibition values. The SOD activity levels are expressed as µmol/min/mg protein.

#### LPO

The LPO in gill and digestive gland tissue homogenates was determined via absorbance generated by TBARS based on adapted methods of Bouskill et al. (2006). Sample homogenates (40 µl) were added to wells of a 96‐well plate in triplicate to which 10 µl of 1 mol/L butylated hydroxytoluene solution, 140 µl of phosphate‐buffered saline (1 mmol/L, pH 7.4), 50 µl of trichloroacetic acid (50% w/v), and 75 µl of thiobarbituric acid (1.3 w/v%) were also added, and then the plates were incubated for 60 min. Products of LPO including malonyldialdehyde formed were estimated at 530 and 630 nm (Spectra Max M5) and correlated with tetraethoxypropane standards (0.5–1 × 10^6^ nM). The TBARS levels were expressed as nmol/mg protein.

#### Comet assay

Hemocytes and gill cells were analyzed for genotoxicity using the comet assay. The single‐cell gel electrophoresis or comet assay was performed according to Singh and Hartl (2012) and Al‐Shaeri et al. (2013). A detailed description of the methodology can be found in the Supporting Information.

### Collection of biodeposits

Mussel biodeposits (feces and pseudofeces) from each treatment group (6 mussels/treatment) were collected at the end of the 21‐day exposure experiment. To do so, seawater from each treatment group (2.4 L) was filtered through glass microfiber filter papers (1.2 µm; Whatman) using a peristaltic pump. Collected biodeposits were rinsed off the filter paper using seawater, and left to settle overnight in 50 ml centrifuge tubes. In cases in which the biodeposits did not settle, a centrifugation step was used to ensure all material remained in the vessel when the seawater (supernatant) was carefully discarded. Biodeposits were then oven‐dried (90 °C) for 4 h and weighed. These samples were then acid‐digested and analyzed for Ti and Cu content using inductively coupled plasma–mass spectrometry (ICP–MS).

### Cu and Ti content analysis using ICP–MS

Mussel tissues were thawed before undergoing digestion using nitric acid (HNO_3_). Briefly, tissues were first digested in 1 ml 70% trace analysis grade HNO_3_ for 2 h in a water bath set to 80 °C, after which they were diluted using 5% HNO_3_ to a final volume of 5 ml. Samples were then analyzed for Cu and Ti using a Thermo Scientific X Series 2 operating under a radiofrequency power of 1400 W, using a coolant, nebulizer, and gas flow of 13 L/min argon, 0.7 L/min argon, and 0.78 L/min argon, respectively. The equipment was fitted with a Burgener nebulizer and a cyclone spray chamber type and was operating under a dwell time of 10 min and 50 sweeps.

Samples were spiked with indium (In) and iridium (Ir), which acted as internal standards. A 92% and 89% recovery rate was achieved for Ti and Cu according to analysis of samples spiked with known concentrations of TiO_2_ and CuO ENMs. The Ti and Cu levels measured in µg/L according to ICP–MS were adjusted according to final volume (5 ml) and wet weight of tissue in grams, and values are presented in mg/g tissue (wet wt) or µg/g tissue (wet wt) where appropriate.

Prior to elemental analysis, dried biodeposits were digested in 2 ml HNO_3_ at 80 °C for 4 h in a water bath and left at room temperature overnight for further digestion. Digests were then diluted using 5% HNO_3_ to a final volume of 10 ml. The ICP–MS analysis was then performed using the same equipment and parameters as just outlined. Again, the Ti and Cu levels measured in µg/L according to ICP–MS were adjusted according to final volume (10 ml) and dry weight of biodeposits in grams, and values are presented in mg/g biodeposit dry weight or µg/g biodeposit dry weight, where appropriate.

### Raman mapping

Confocal Raman microspectroscopy analyses were performed on digestive gland and gill tissues of mussels exposed for 21 days to TiO_2_ NMs using an Alpha300 R microscope (WITec) equipped with a 532‐nm laser source, a 600‐g/mm grating, and a charge‐coupled device cooled down to −61 °C. All measurements were conducted using a 63× water immersion objective (W Plan‐Apochromat 63×/1.0; Zeiss). Paraffin‐embedded tissue samples of digestive glands were cut in 5–10‐µm‐thick sections and mounted onto glass slides. After deparaffinization, water‐mounted mussel tissue was scanned with a laser power of approximately 35 mW at 532 nm. Raman spectra were collected pixel‐wise with an integration time of approximately 0.07 s. Acquired spectra were processed using the Project FOUR PLUS software (WITec).

### Statistical analysis

Data were analyzed for normality and homogeneity of variance using the Shapiro–Wilk test. One‐way analysis of variance and Tukey tests were used to detect significant differences between exposures for DNA damage, SOD, TBARS, and accumulation data (IBM SPSS®) with graphs produced using RStudio. Dose–response curves for in vitro cytotoxicity screening were fitted using a four‐parameter logistic nonlinear regression model in GraphPad Prism® and used to calculate EC50 values.

## RESULTS

### NM physicochemical characterization

Stable suspensions of TiO_2_ core, PEG, COOH, and NH_3_ coated ENMs dispersed in deionized water (2 mg/ml) had hydrodynamic sizes of 15.41, 121, 122.3, and 32.9 nm, respectively. Thereafter, working concentrations were prepared in seawater or HBSS+ (200 µg ENM/ml, 16 °C). These suspensions were characterized using DLS (Tables 1 and 2). Aggregation/agglomeration occurred for all particle suspensions in seawater, and a similar phenomenon was seen in HBSS+ medium. The high polydispersity index (PDI) values for TiO_2_‐PEG and COOH indicate a very broad particle size distribution in suspensions (Table 1). Aggregates in these suspensions of TiO_2_‐PEG and COOH with mean size intensity values of 141 and 196 nm, respectively, were measured; however, these suspensions are also likely to have much larger aggregates according to the overall higher *Z*‐average values recorded. The TiO_2_‐NH_3_ suspensions were characterized by generally large‐sized aggregates (mean intensity of 952 nm and a *Z*‐average of 2090 nm). The TiO_2_ core suspensions showed a narrower size distribution (PDI value of 0.117, suggesting a relatively monodisperse suspension), with aggregates of mean size intensity of approximately 1300 nm, and this is consistent with the *Z*‐average values recorded.

**Table 1:** Hydrodynamic size distribution of TiO_2_ and CuO engineered nanomaterial (ENM) suspensions prepared in seawater for acute and chronic exposures and HBSS+ for hemocyte in vitro screening assays^a^

	Seawater			HBSS+		
Nanomaterial	PDI	*Z*‐average (nm ± SD)	Intensity (d.nm ± SD)	PDI	*Z*‐average (nm ± SD)	Intensity (d.nm ± SD)
TiO_2_ core	0.117	1289 (±109)	1375 (±140)	0.553	2832 (±271)	1765 (±468)
TiO_2_ PEG	1	3592 (±680)	141 (±49)	0.834	557 (±193)	184 (±17)
TiO_2_ COOH	0.983	1373 (±60)	196 (±17)	0.607	481 (±155)	122 (±31)
TiO_2_ NH_3_	0.386	2090 (±186)	952 (±89)	0.243	127 (±5)	150 (±30)
CuO core	0.538	3358 (±752)	1530 (±575)	0.221	1286 (±85)	1139 (±253)
CuO PEG	0.165	2792 (±122)	1461 (±492)	0.72	756 (±62)	310 (±40)
CuO COOH	0.371	3843 (±209)	1137 (±821)	0.246	1562 (±68)	987 (±224)
CuO NH_3_	0.343	1767 (±140)	1305 (±97)	0.13	975 (±99)	1039 (±95)

^a^CuO ENM stock dispersions in deionized water had measured hydrodynamic sizes for CuO core, PEG, COOH, and NH_3_ of 562.9, 669.3, 815, and 567.4 nm, respectively.

TiO_2_ = titanium dioxide; CuO = copper(II) oxide; HBSS+ = Hanks' balanced salt solution; PEG = polyethylene glycol; COOH = carboxyl; NH_3_ = ammonia; CuSO_4_ = copper(II) sulfate; PDI = polydispersity index; d.nm = diameter in nanometers.

**Table 2:** Median effect concentration (±SEM) values calculated from dose–response curves and confidence intervals (CIs) following a 2‐h exposure of mussel hemocytes to uncoated CuO core engineered nanomaterials (ENMs) and coated CuO ENMs

Nanomaterial	EC50 (µg Cu/ml)	95% CI
CuO core	17.14 (±3.1)	7.87–37.30
CuO PEG	2.11 (±1.6)	0.68–6.54*
CuO COOH	3.23 (±0.5)	2.44–4.28*
CuO NH_3_	6.33 (±1.7)	4.27–9.37
CuSO_4_	3.85 (±0.9)	1.77–8.37

* Significant difference from CuO core treatment because confidence intervals do not overlap.

EC50 = median effect concentration; CuO = copper(II) oxide; PEG = polyethylene glycol; COOH = carboxyl; NH_3_ = ammonia; CuSO_4_ = copper(II) sulfate.

Working suspensions experienced further aggregation in seawater and HBSS+. The CuO ENM suspensions were characterized by similar size distributions, irrespective of the different coatings, in seawater, with aggregates ranging in mean size intensity between 1137 and 1530 nm. The higher *Z*‐average values measured compared with size intensity values would also suggest some degree of larger aggregate formation in the suspensions. Visible sedimentation of CuO ENM particles out of suspension over time was also evidenced. The CuO ENM suspensions prepared in HBSS+ exhibited distinct aggregation behavior (*Z*‐average and mean intensity values) compared with seawater suspensions and among the different coated CuO ENMs. Smaller aggregates were measured in CuO‐PEG ENM suspensions, with mean intensity values of 310 nm compared with mean intensity values of 1139, 987, and 1039 nm measured in CuO core, COOH, and NH_3_ ENMs suspensions. The PDI values were varied, with CuO‐PEG in seawater and CuO‐NH_3_ in HBSS+ suspensions showing lower PDIs, indicating more monodispersity.

### In vitro cytotoxicity screening studies

The TiO_2_ ENMs did not produce any lysosomal membrane permeabilization in hemocytes following 2 h of exposure to core or coated ENMs (200 µg or more ENM/ml; data not shown). In contrast, all CuO ENMs produced concentration‐dependent lysosomal permeabilization. The coated particles produced effects at lower concentrations compared with the core ENMs. The EC5O values calculated for treatments are presented in Table 2. There was no evidence of assay interferences by the ENMs (no apparent absorption of neutal red dye to the ENMs and no fluorescence quenching; data not shown).

According to the calculated EC50 values following 2 h of exposure, coated CuO ENMs were found to be more toxic. Cytotoxicity was seen at lower exposure concentrations, and the calculated EC50 values, although similar for CuO‐PEG and COOH, were much lower than for CuO core ENMs. The confidence interval ranges also indicated significant difference in response to the PEG and COOH coated CuO compared with the core CuO. The EC50 for CuSO_4_, included as a positive control in assays, was consistent with values reported for hemocyte responses to Cu ion in bivalve mussels (24 h EC50: neutral red uptake assay: 4.25 µg Cu/ml). In addition, the EC50 value reported for uncoated CuO ENMs (aggregates 197 and 810 nm) was 8.14 µg Cu/ml (Katsumiti et al., 2018).

### Acute effects: Cellular level genotoxicity

**Figure 2:**
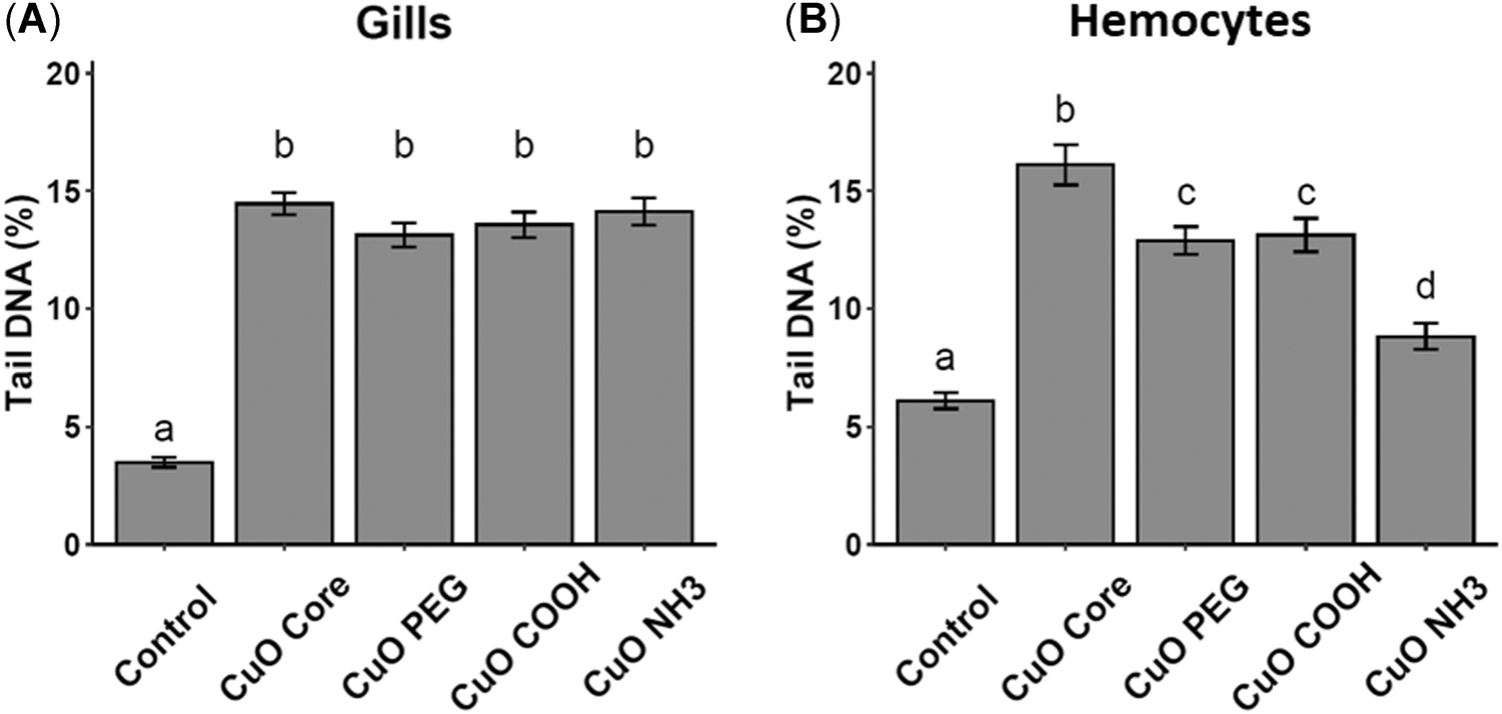
DNA damage measured in gills (**A**) and hemocytes (**B**) of *Mytilus* spp. exposed to copper(II) oxide (CuO) engineered nanomaterials (20 µg/L) for 48 h (±SEM, *n* = 6). Bars with different letters denote significant difference between treatments (*p* < 0.05). PEG = polyethylene glycol.

Genotoxic effects (48 h exposure) in hemocytes and gill cells of mussels exposed to CuO ENMs were analyzed (genotoxicity analysis for TiO_2_ ENMs was not performed due to lack of effects at Tier 1). An exposure concentration was chosen (20 µg/L) taking into consideration the EC50 values and using 1/10 of the lowest value calculated and reducing this concentration again by a factor of 10 to allow for long‐term sublethal tests to be performed. All the CuO ENMs tested produced genotoxic effects according to an increase in % tail DNA measured in gill cells and hemocytes of CuO ENM‐exposed mussels (Figure 2A and B). No differences in measured genotoxicity was recorded for the different CuO ENMs. However, in hemocytes, higher DNA damage was measured in CuO core ENM‐exposed mussels compared with the coated CuO ENMs (Figure 2B), whereas the lowest DNA damage was measured for CuO NH_3_.

### Acute effects: Tissue biomarkers of oxidative stress

**Figure 3:**
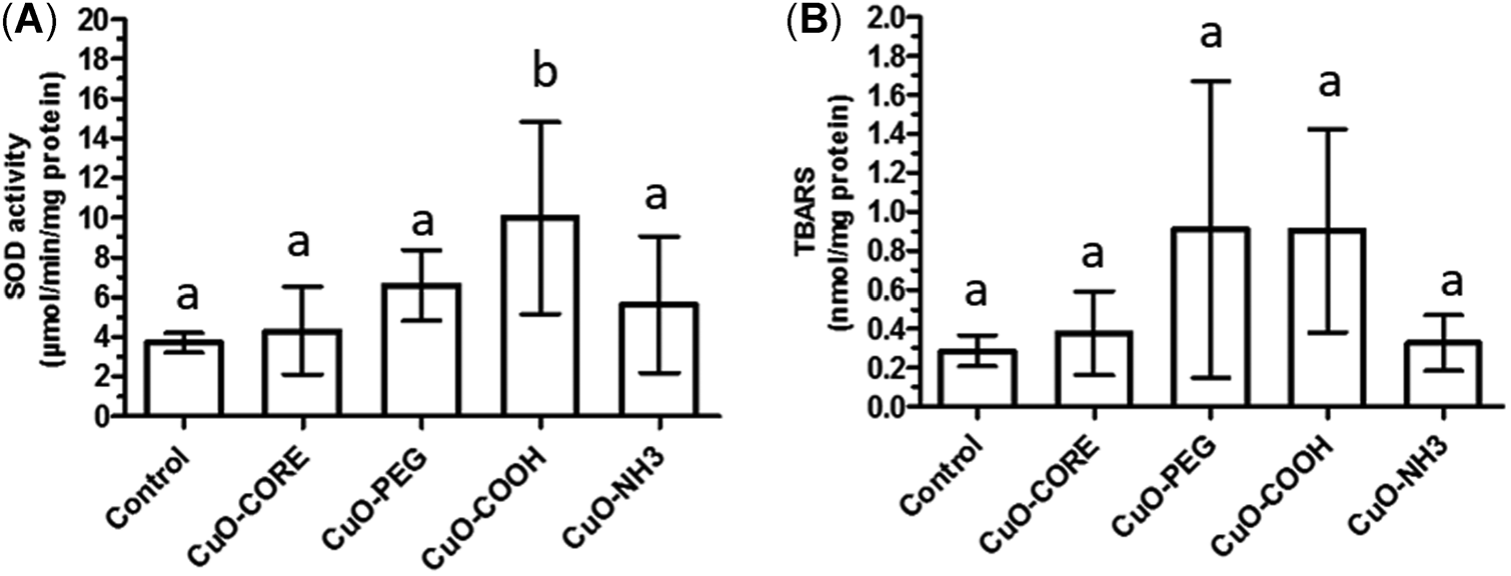
Superoxide dismutase (SOD) activity levels (**A**) and thiobarbituric acid reactive substance (TBARS) levels (**B**) in gill tissue of *Mytilus* spp. in control and copper(II) oxide (CuO) engineered nanomaterial exposure groups. Data are mean ± SEM (*n* = 6). Bars with different letters denote significant difference between treatments (*p* < 0.05). PEG = polyethylene glycol.

Following 48 h of exposure to CuO‐COOH ENMs, there was a significant increase in activity levels of SOD (*p* < 0.05; Figure 3A). Levels of TBARS were also elevated in gill tissues of mussels exposed to CuO‐COOH (albeit not significant due to large deviations in levels measured; Figure 3B). Levels of TBARS were also increased in CuO‐PEG–exposed mussel gill tissue, but again, a very large standard deviation was seen.

### Tissue biomarkers of oxidative stress: 7‐, 14‐, and 21‐day sub/chronic effects

Gill and digestive gland tissues of CuO and TiO_2_ ENM‐exposed mussels were analyzed for biomarkers of sublethal effects associated with oxidative stress following long‐term exposures (7–21 days) to 20 and 300 µg/L, respectively.

#### CuO ENMs

**Figure 4:**
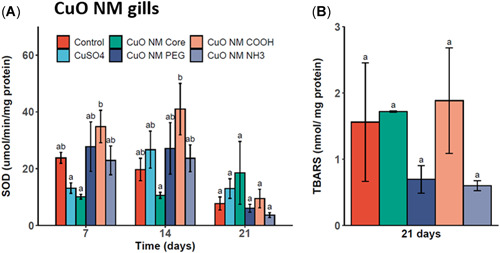
Superoxide dismutase (SOD) levels in gill tissue homogenates of mussels exposed to copper(II) oxide (CuO) engineered nanomaterials (ENMs) and copper(II) sulfate (CuSO_4_) for 7, 14, and 21 days (**A**) and lipid peroxidation (thiobarbituric acid reactive substance [TBARS]) following CuO ENM exposure for 21 days (**B**). Data are mean ± SEM (*n* = 3). Bars with different letters denote significant difference between treatments (*p* < 0.05).

A significant increase in SOD activity was measured in the gill tissues of mussels exposed to CuO‐COOH ENMs compared with CuSO_4_ and CuO core exposures after 7 days and CuO core after 14 days (Figure 4A). This effect was specific for this treatment group, with no significant increases in activity levels measured in the gill tissues of the other coated CuO ENM‐exposed mussels. Exposure to CuO core ENMs and CuSO_4_ resulted in a decrease in SOD activity levels in gill tissues at day 7. At day 14, activity levels in CuSO_4_ gill tissues increased to similar levels as measured in the control, but the activity in CuO core gill tissues remained lower. At 21 days the SOD activities in all treatment groups were significantly similar to one another and controls. In addition, TBARS levels were not significantly different from control levels in 21‐day exposed mussels. The TBARS levels measured at day 21 (as a biomarker of oxidative imbalance) indicated no significant increases in LPO products in gill tissues for any of the CuO ENM treatments (Figure 4B).

**Figure 5:**
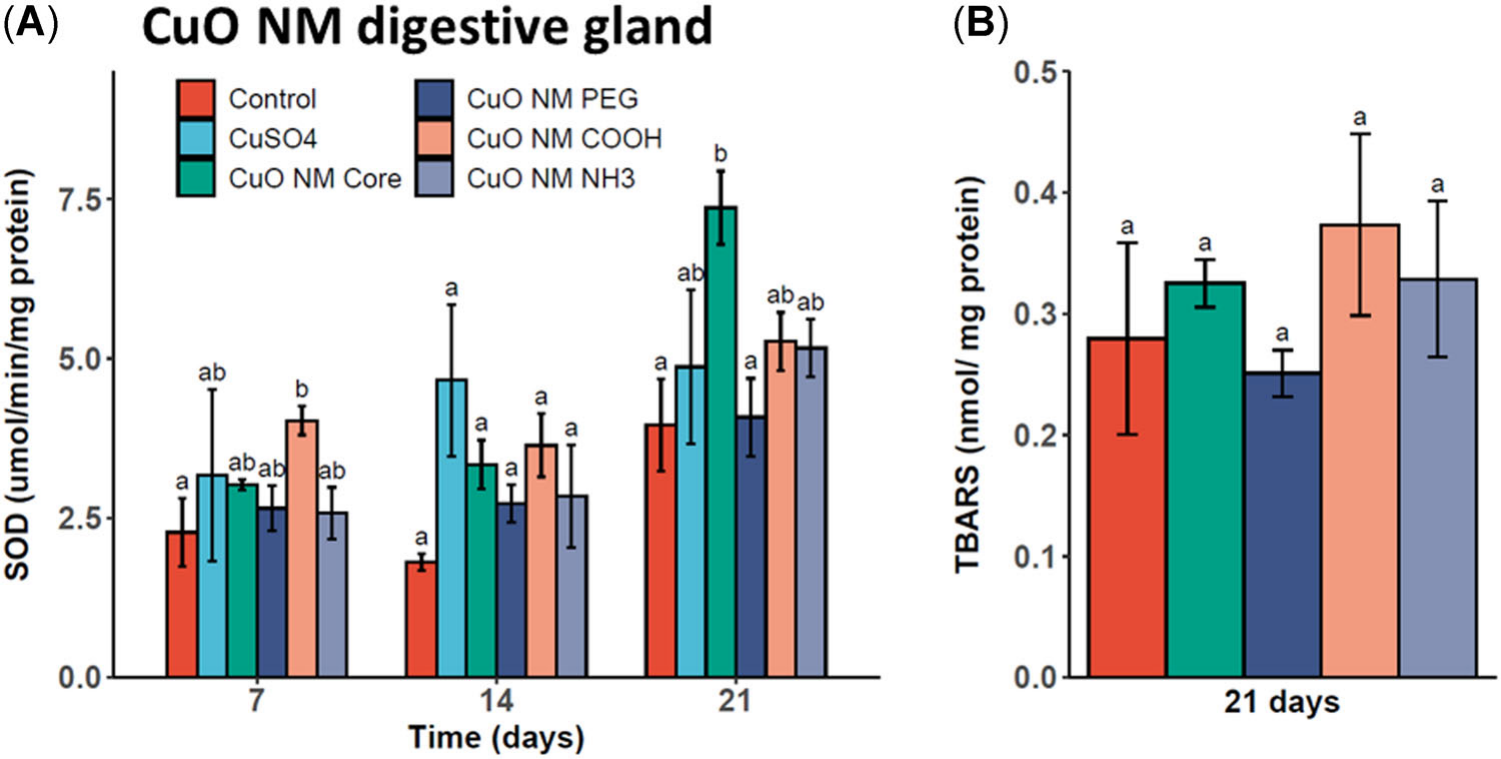
Superoxide dismutase (SOD) levels in digestive gland homogenates of *Mytilus edulis* exposed to copper(II) oxide (CuO) engineered nanomaterials (ENMs) and copper(II) sulfate (CuSO_4_) for 7, 14, and 21 days (**A**) and lipid peroxidation (thiobarbituric acid reactive substance [TBARS]) following CuO ENM exposure for 21 days (**B**). Data are mean ± SEM (*n* = 3). Bars with different letters denote significant difference between treatments at each time point (*p* < 0.05). PEG = polyethylene glycol.

Significant increases in SOD activity levels were also measured in digestive gland tissues of CuO‐COOH–exposed mussels compared with controls at day 7 (Figure 5A). At day 14, no significant differences were measured, but at day 21, significant increases in SOD activity levels were evidenced in CuO core ENM‐exposed mussel digestive gland tissues compared with controls and CuO‐PEG. This effect did not lead to significant increases in digestive gland LPO in CuO core ENM‐exposed mussels or in the other treatment groups after 21 days (Figure 5B).

#### TiO_2_ ENMs

**Figure 6:**
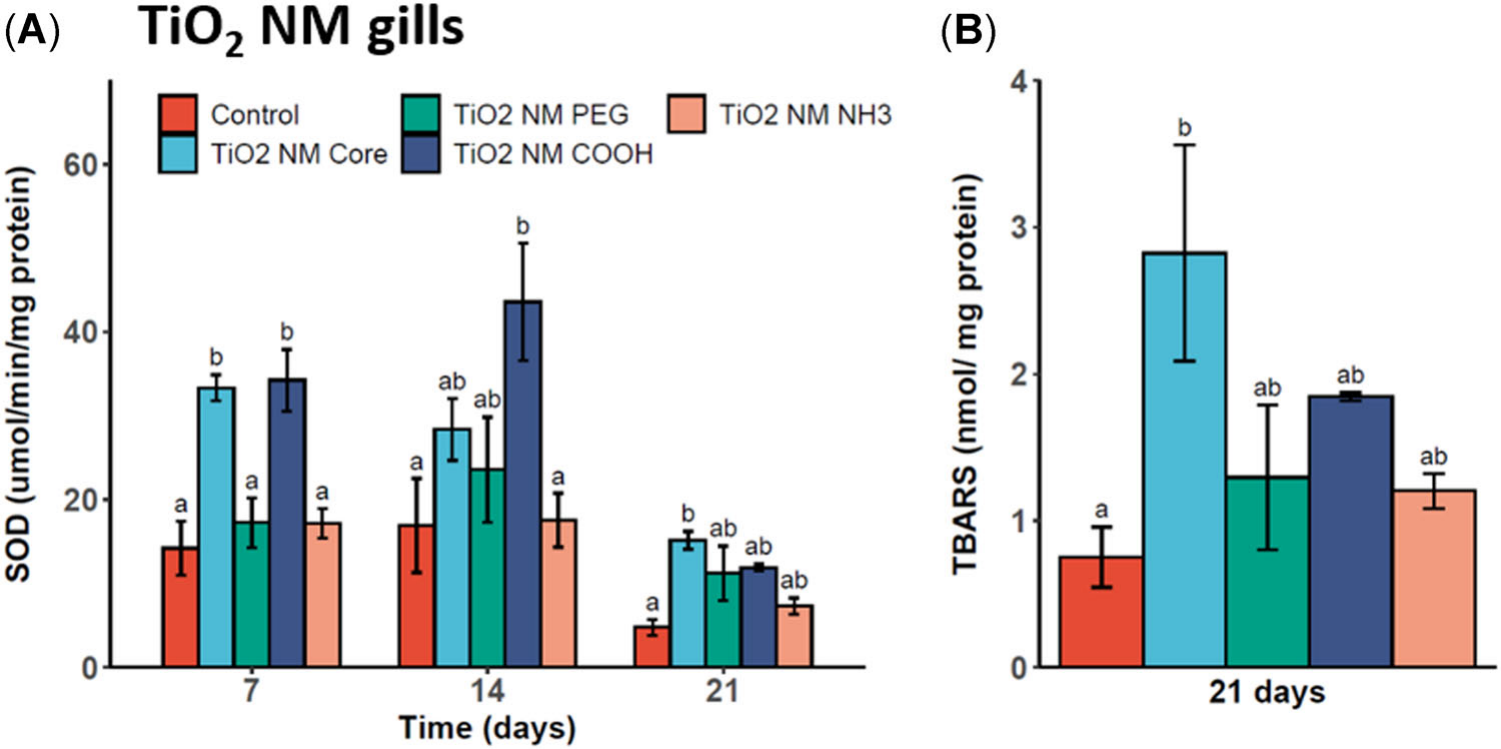
Superoxide dismutase (SOD) levels in gill tissue homogenates of mussels exposed to titanium dioxide (TiO_2_) engineered nanomaterials (ENMs) for 7, 14, and 21 days (**A**) and lipid peroxide (thiobarbituric acid reactive substance [TBARS]) following TiO_2_ ENM exposure for 21 days (**B**). Data are means ± SEM (*n* = 3). Bars with different letters denote significant difference between treatments at each time point (*p* < 0.05). PEG = polyethylene glycol.

Despite a lack of toxicity in Tier 1 testing, potential subchronic effects were investigated in TiO_2_ NM‐exposed gill and digestive gland tissues (Figure 6). Distinct and significant increases in SOD activity levels in gill tissues exposed to the TiO_2_ core and TiO_2_‐COOH NMs for 7 days were seen (Figure 6A). The levels of activity measured were significantly elevated (*p* < 0.05) compared with control and other coated TiO_2_ NMs. After 14 days of exposure, levels of SOD activity in TiO_2_‐COOH–exposed mussels remained elevated from the control and NH_3_‐coated CuO NM group levels. At day 21, all SOD activities measured were elevated from the control, with a significant increase measured in TiO_2_ core exposed mussel gill tissues. Also, significantly higher levels of TBARS were measured in TiO_2_ core ENM‐exposed mussel gill tissues compared with control mussel levels after 21 days.

**Figure 7:**
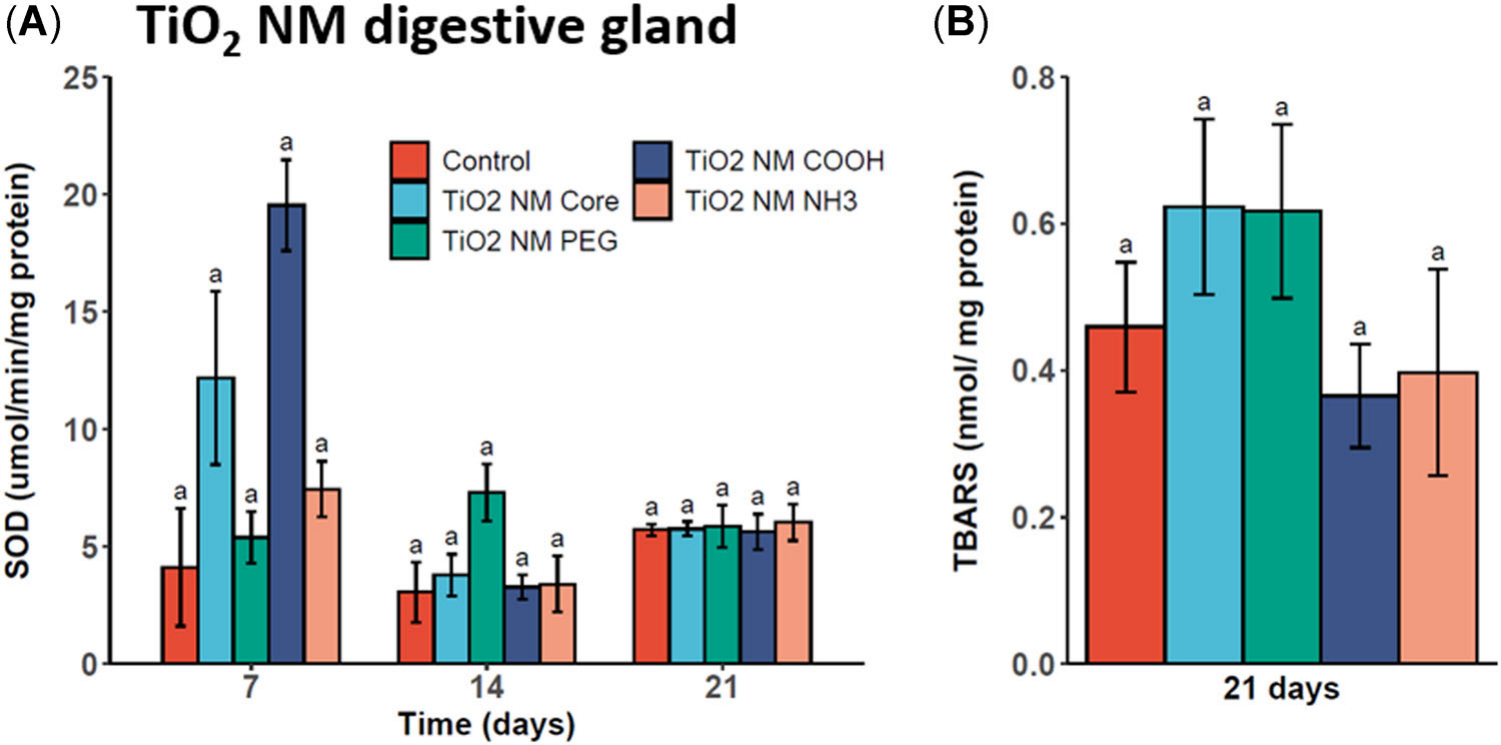
Superoxide dismutase (SOD) levels in digestive gland tissue homogenates of mussels exposed to titanium dioxide (TiO_2_) engineered nanomaterials (ENMs) for 7, 14, and 21 days (**A**) and lipid peroxidation (thiobarbituric acid reactive substance [TBARS]) following TiO_2_ ENM exposure for 21 days (**B**). Data are means ± SEM (*n* = 3). Bars with different letters denote significant difference between treatments at each time point (*p* < 0.05). PEG = polyethylene glycol.

In digestive gland tissues, elevated levels of SOD activity were measured in TiO_2_ core and TiO_2_‐COOH ENM‐exposed mussels following 7 days (Figure 7A); however, these differences were not found to be significant. After 14 and 21 days, there was no significant difference in measured SOD activity levels for any of the treatment groups compared with the unexposed control. In addition, TBARS levels remained unchanged from control levels in digestive gland tissues following 21 days of exposure to all TiO_2_ ENMs (Figure 7B).

### ENM bioaccumulation and biological fate

#### Cu and Ti levels in tissues

**Figure 8:**
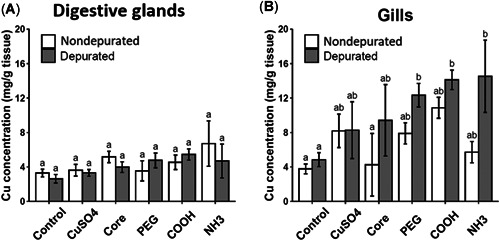
Copper (Cu) levels in digestive gland (**A**) and gill (**B**) tissues of mussel exposed to copper(II) oxide (CuO) engineered nanomaterials (ENMs) for 21 days (nondepurated) and after being allowed to depurate for 24 h (depurated). Data are means ± SEM (*n* = 3). Bars with different letters denote significant difference between treatments (*p* < 0.05). PEG = polyethylene glycol.

**Figure 9:**
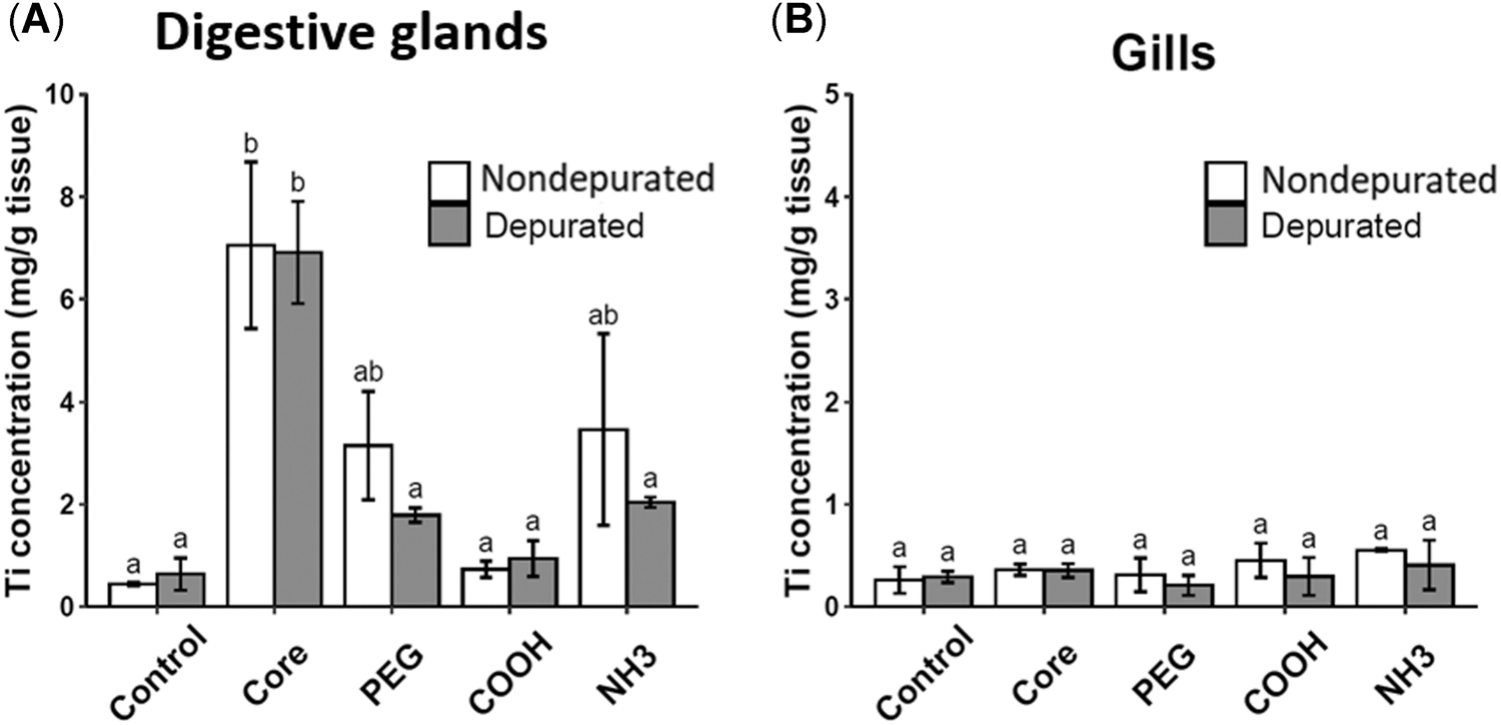
Titanium (Ti) levels in digestive gland (**A**) and gill (**B**) tissues of mussels exposed to titanium dioxide (TiO_2_) engineered nanomaterials (ENMs) for 21 days (nondepurated) and after being allowed to depurate for 24 h (depurated). Data are means ± SEM (*n* = 3). Significant differences (*p* < 0.05) between treatments are represented by dissimilar letters. PEG = polyethylene glycol.

Digestive gland and gill tissues of the respective CuO and TiO_2_ ENM treatment groups were analyzed for significant uptake of Cu and Ti following 21‐day exposure. In addition, a 24 h depuration step was used, and thereafter levels in tissues were compared with those measured in nondepurated mussels (Figures 8 and 9).

#### CuO ENMs

There was no significant uptake of Cu in the digestive gland of mussels exposed to CuO ENMs or CuSO_4_ for 21 days (Figure 8A). In gill tissues, Cu uptake in nondepurated mussels took the following pattern: CuO‐COOH > CuSO_4_ > CuO‐PEG > CuO‐NH_3_ > CuO core > control. Levels of Cu were higher in depurated gills compared with nondepurated gills for each exposure; however, none of these increases were significant (*p* > 0.05). Depurated mussel gills exposed to CuO‐PEG had significantly higher Cu levels compared with depurated and nondepurated controls (*p* = 0.44 and 0.048, respectively), as did COOH‐exposed mussels (*p* = 0.009 and *p* = 0.024) and NH_3_‐exposed mussels (*p* = 0.017 and 0.041). The biggest increase observed was for CuO‐NH_3_ (Figure 8B). The highest levels of Cu were also measured in biodeposits collected from the CuO‐NH_3_ ENM‐exposed mussels after 24 h of depuration (Supporting Information, Figure S2).

#### TiO_2_ EMNs

Although noticeable Ti increases were observed in digestive glands exposed to TiO_2_ core, PEG, and NH_3_, significant differences were only detected for TiO_2_ core (Figure 9A). Nondepurated core CuO ENM‐exposed tissues recorded significantly greater Ti concentrations than the control (nondepurated, *p* = 0.001 and depurated, *p* = 0.001), COOH (nondepurated, *p* = 0.002 and depurated, *p* = 0.003), and the depurated tissues of PEG (*p* = 0.11) and NH_3_ (*p* = 0.040) exposed digestive glands. Despite decreases in Ti concentration following depuration for TiO_2_‐PEG and ‐NH_3_ exposures, Ti concentrations remained high in TiO_2_ core exposures. Depurated core CuO ENM‐exposed tissues had significantly greater Ti concentrations than the control (nondepurated, *p* = 0.001 and depurated, *p* = 0.006), COOH (nondepurated, *p* = 0.002 and depurated, *p* = 0.003), and depurated tissues of PEG (*p* = 0.014) and NH_3_ (*p* = 0.048) exposed digestive glands.

There was no significant uptake of Ti in gill tissues of TiO_2_ ENM‐exposed mussels after 21 days of exposure, nor were there any increases in levels measured in depurated mussels (Figure 9B). Increased levels of Ti were measured in the biodeposits collected from all TiO_2_ ENM‐exposed mussels left to depurate for 24 h (Supporting Information, Figure S2).

### Raman microscopy

**Figure 10:**
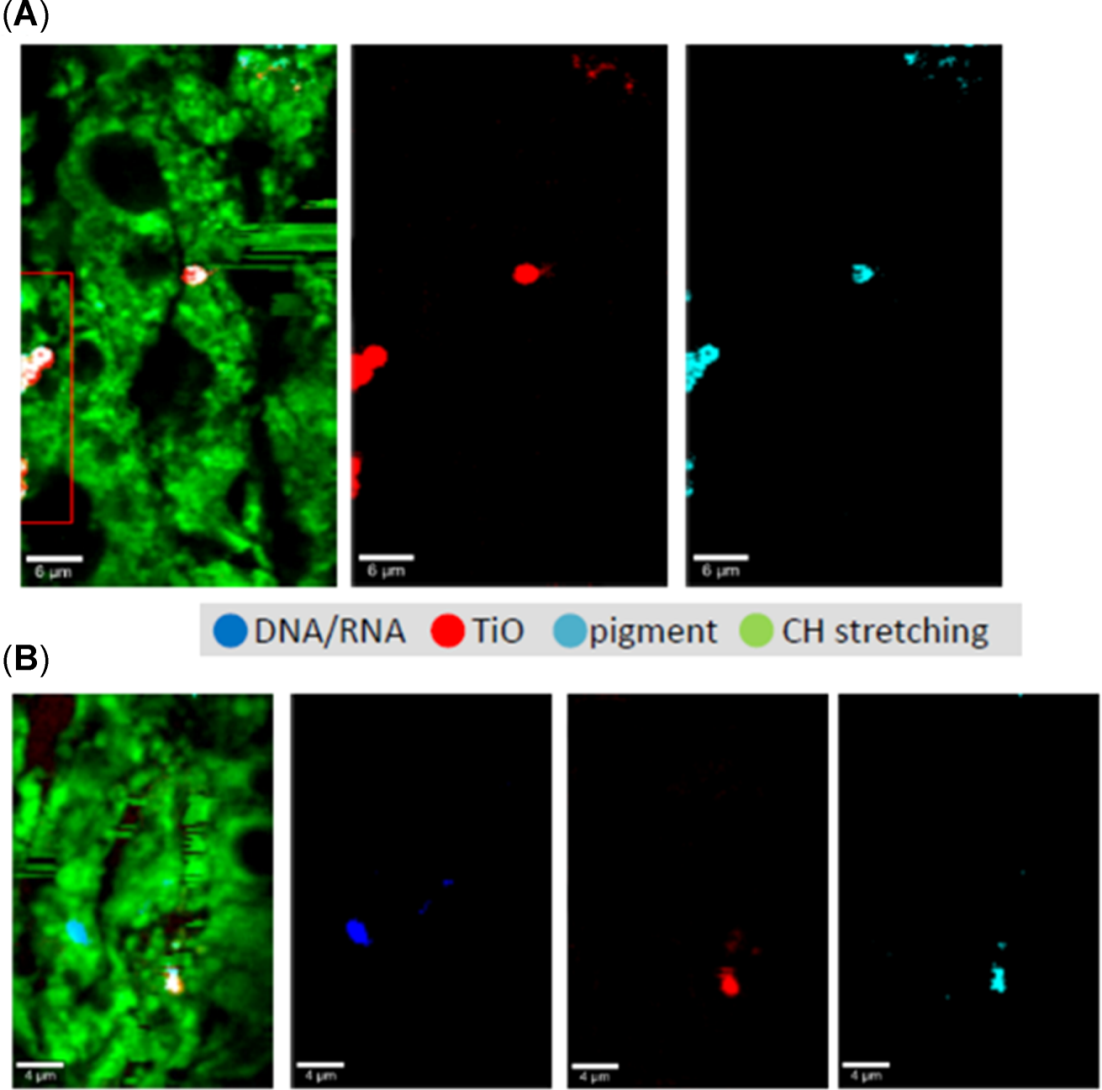
Raman mapping of digestive gland tissue of mussels following 21‐day exposure to (**A**) titanium dioxide (TiO_2_) core engineered nanomaterials (ENMs) and (**B**) TiO_2_ NH_3_ ENMs. Images have incorporated all signals including C–H stretching from background carbon and hydrogen in tissues (green), DNA/RNA (dark blue), and the signal from TiO_2_ (red) and a fluorescent pigment (light blue). Individual signals from TiO_2_ and the fluorescent pigment, obtained by removing the background C–H stretching, are also presented. Scale bars are 6 and 4 µm in (**A**) and (**B**), respectively. Additional images are presented in the Supporting Information (Figure S3).

Raman confocal microscopy was used to analyze tissues (gills and digestive glands) after 21 days of exposure to TiO_2_ ENMs. No Ti could be detected in gill tissues of any of the treatment groups, nor in the digestive gland of TiO_2_‐COOH or TiO_2_‐PEG ENM‐exposed mussels. Areas with concentrated levels of Ti were detected in the digestive glands of TiO_2_ core and NH_3_ ENM‐exposed mussels (Figure 10). Interestingly, pigments were observed in close association with accumulated TiO_2_ ENMs. We postulate that the observed pigmentation is potentially fluorescent chlorophyll from algae fed to mussels, or lipofuscein granules that are overproduced in oxidized tissues. Although it was not possible to perform quantitative analysis of pigments to confirm whether they were lipofuscein granules and whether they increased as a result of TiO_2_ exposure, a clear relationship between the two was evident and would make an interesting investigation in future research.

Raman mapping was not used to investigate CuO ENM accumulation due to the difficulty in discriminating Cu signal from ENMs in the presence of likely high levels of the Cu‐containing hemocyanin proteins present in the blood of many molluscs.

## DISCUSSION

In the present study we developed an ITS for ecotoxicity assessment (ITS‐ECO) to characterize the hazard and fate of ENMs in the marine compartment using a single species, mechanistic focused, tiered testing approach. Specific ENM ecotoxicological testing frameworks are scarce (Hund‐Rinke et al., 2015), particularly for marine environmental compartments. Such frameworks, which incorporate screening‐level assessments, are potentially a good strategy to avoid unnecessary higher tier testing. The development of testing strategies and the specific data they generate will feed into the IATAs of the future (Stone et al., 2020).

In vitro assessment using hemocytes (as cells integral to the defence against contaminants) allowed the screening and, when possible, ranking of TiO_2_ and CuO ENM hazards according to their EC50 values in an high‐throughput approach within Tier 1. The use of hemocytes in toxicity assessment of legacy and emerging contaminants including ENMs has been explored in the past (Katsumiti et al., 2018); however, their inclusion within the ITS‐ECO as a tool for hazard screening demonstrates their novelty and high potential for future testing strategies as an alternative to in vivo testing. Based on high‐throughput dose‐dependent effects recorded for CuO ENMs, the comet assay was used to investigate whether acute, in vivo exposure elicited genotoxic effects. All CuO ENMs were found to cause DNA damage in gills (no coating‐specific effects) and hemocytes (highest DNA damage in CuO core ENM‐exposed mussels) at exposure concentrations of 20 µg/L. Due to the lack of cytotoxicity in hemocytes, genotoxic effects were not assessed for TiO_2_. At higher exposure concentrations of 200 and 1000 µg/L or more, genotoxic effects of TiO_2_ ENMs in gill and digestive gland cells and hemocytes of mussels, respectively, have been shown (Girardello et al., 2016; Kukla et al., 2018).

Both short‐ and long‐term exposures were employed in Tier 2 testing to account for the transient nature of antioxidant enzymes and the increasing need to assess chronic effects in testing strategies (Selck et al., 2016). Whereas increases in SOD were recorded in gill and digestive gland tissues exposed to CuO and TiO_2_ ENMs, LPO was only detected in gill tissues exposed to TiO_2_ core ENMs.

In Tier 3 bioaccumulation studies, CuO ENMs predominantly accumulated in gill tissues, whereas TiO_2_ ENMs were detected to a greater extent in digestive glands. Although traditional bioaccumulation studies employ fish, invertebrate species such as bivalves could be regarded as particularly relevant for ENM testing, and their use in an alternative approach to bioaccumulation testing for a worst‐case scenario (due to specific high filtering capacity) with a focus on prediction and a reduction in vertebrate testing should be in a tiered approach.

The results from the ITS‐ECO developed and employed for hazard and fate assessment of the core and coated TiO_2_ and CuO ENMs in the present study are discussed in the next section.

### TiO_2_ and coated TiO_2_ ENM hazard and fate assessment using ITS‐ECO approach

An absence of lysosomal stability effects in Tier 1 was witnessed after 2 h of TiO_2_ ENM exposure (at concentrations of 200 µg/ml or less) and suggests that TiO_2_ ENMs, regardless of the presence or absence of coatings, could be classified as having low hazard potential. This is consistent with short‐term exposure studies in vitro performed in other mussel species in which the 2 h EC50 values calculated were in the range of 234–518 µg/ml (TiO_2_ anatase 32 nm, 45 m^2^/g) or were indeterminable for some species (no reduction in viability at concentrations up to 1000 µg/ml; Pikula et al., 2020). Hemocytes have also been used in primary cell cultures, which allow a longer exposure duration and have shown 24 h EC50 values in the range of 21–43 µg Ti/ml (Katsumuti et al., 2014). It remains possible that longer term hemocyte exposure may have led to significant effects. Thus a 24 h cellular screening may also be incorporated in the ITS‐ECO (provided that primary cell culture and maintenance of cell viability can be established). Dissolution is not expected for these materials either in the hemocyte culture medium (HBSS+) used in cytotoxicity screenings or in seawater due to the low/nondetectable dissolution rate of less than 0.01 μg/min (see the Supporting Information, Table S1).

Tier 2 testing was prioritized for TiO_2_ ENMs (due to low, acute genotoxicity) to determine whether longer exposure elicited oxidative stress‐related effects. In the present study significant increases in SOD activity and TBARS levels in digestive gland and gill tissues of TiO_2_ core and COOH‐ENM‐exposed mussels were recorded. In previous studies, hematoxylin and eosin staining has shown an influx of hemocytes along filaments and acidophilic mucous cells in the apex of the filament in TiO_2_ ENM‐exposed mussels (D'Agata et al., 2014). Further histopathological effects have been shown in digestive glands of marine oysters following 14 days of exposure (0.59 ± 0.13 mg/L; Xia et al., 2017). Increases in SOD activity suggest that mussels activate antioxidant defence systems to prevent cellular damage in gills and digestive tissues following TiO_2_ ENM exposure.

Digestive glands were the major site of Ti uptake following TiO_2_ ENM exposure in Tier 3, as witnessed in other studies (D'Agata et al., 2014). High Ti levels in the digestive gland of TiO_2_ core exposed mussels did not decrease after 24 h of depuration. Although SOD increases were evident after 7 days in digestive gland, levels did not increase following longer exposures, potentially indicative of the antioxidant system becoming overwhelmed or an inability to regulate. Oxidative stress responses in digestive gland tissues following acute exposures have been shown for other TiO_2_ ENMs including the Joint Research Centre representative nanomaterial NM 105 (P25 aeroxide; Barmo et al., 2013). However, there is also evidence that mussels can repair TiO_2_ ENM damage and oxidative effects following a recovery period (Huang et al., 2018).

Compromised clearance may lead to potential biomagnification of Ti in higher trophic levels and present a potential risk for humans. Recent studies have shown TiO_2_ ENM contents of mussels to be higher than those in seawater (Xu et al., 2020), indicating an accumulation of TiO_2_ ENMs.

Bioaccumulation factors serve as endpoints for bioaccumulation assessment and to compare bioaccumulation potential according to defined thresholds. Although most commonly reported for fish, other relevant target organisms can be used, and mussels have been proposed in bioaccumulation testing approaches (Handy et al., 2018; Kuehr et al., 2021). Care must be taken, however, to differentiate between the body burden resulting from ENMs attached to the animal's surface or simply ingested and those incorporated and bioaccumulated (Petersen et al., 2019). This is particularly relevant for mussels, which have an open circulatory system and could lead to overestimations of bioaccumulation potential. Specific attention has been given to this in the approach to bioaccumulation assessment in mussels proposed by Kuehr et al. (2021), which includes the calculation of a time‐weighted residual capacity bioconcentration factor (BCF_TWRC_) based on the concentration that remains following an extended depuration phase divided by the exposure concentration (BCF_TWRC_ would classify a substance as “bioaccumulative” if it was higher than 2000 and “very bioaccumulative” if it was higher than 5000; Kuehr et al., 2021).

In the present study, bioaccumulation factors can be calculated in digestive gland tissues by dividing ENM tissue concentrations (*C*_t_; mg/kg wet wt) by the concentration in the water (*C*_W_; mg/L) according to the equation *C*_t_/*C*_W_ (OECD test guideline 305; 2012). Bioaccumulation factors for core, PEG‐, and NH_3_‐coated TiO_2_ ENMs of 23 666, 10 333, and 12 666, respectively, can be calculated. No significant uptake from background control levels were measured in TiO_2_‐COOH mussels. This suggests a coating effect leading to a reduction in uptake and also influencing excretion, with higher levels of excretion of Ti for the different coated TiO_2_ ENMs during depuration (elimination half‐life of  ≤1 day). Conversely, following 24 h of depuration, Ti levels in TiO_2_ core ENM‐exposed mussel digestive glands remained high (elimination half‐life >1 day). This highlights the distinct effects, behaviors, and fate of core and coated ENMs that will need to be assessed, respectively. for their hazard, fate, and overall risk. The high levels of uptake and lack of excretion in mussels exposed to TiO_2_ core ENMs following 24 h of depuration warrant an extension in the depuration phase for bioaccumulation potential classification. However, what is clear is that a certain degree of clearance is seen already after 24 h of depuration for the coated TiO_2_ ENMs and thus they appear to be less bioaccumulative than the core TiO_2_ ENMs.

Raman confocal microscopy detected Ti in the digestive glands of mussels exposed to TiO_2_ ENMs after 21 days (core and NH_3_). Interestingly Ti signals were associated with pigments postulated to be algae or lipofuscin (also evident in control tissues). It is unlikely that algae (supplied on day 15) would remain in the digestive tract unless strong absorption to accumulated TiO_2_ occurred. Lipofuscins are autofluorescent pigments regarded as end products of LPO (Grune et al., 2004). Interestingly, increases in TBARS levels for LPO products in TiO_2_ core ENM‐exposed digestive glands support the potential presence of these pigments. A significant increase in lipofuscins in the lysosomes of mussels has also been seen by Barmo and colleagues in response to TiO_2_ exposure at concentrations of 1 μg/L or greater (96 h, digestive gland; Barmo et al., 2013). If quantified, lipofuscin may also act as a useful biomarker of oxidative stress, as demonstrated in other organisms (e.g., *Caenorhabditis elegans*; Aan et al., 2013).

### CuO and coated CuO ENM hazard and fate assessment using ITS‐ECO approach

Based on calculated EC50 values, the hazard potentials of CuO ENMs were ranked as: CuO‐PEG > CuO‐COOH > CuO‐NH_3_ > CuO core. The Cu salt tested in parallel showed a similar dose response to CuO‐PEG and ‐COOH, indicating that dissolution of Cu ions potentially contributed to toxicity, or that these materials are equally as cytotoxic as the Cu salt in vitro. The higher dissolution rates reported for CuO‐PEG and ‐COOH (Supporting Information, Table S1) would support the role of Cu ion in the cytotoxic effects seen in vitro and in hazard potential ranking. Following sublethal, acute exposure, genotoxicity at the cellular level in hemocytes and gill tissues was seen for all CuO ENMs, which is consistent with the mussel DNA damage previously reported (Chelomin et al., 2017) and for human cells (Ahamed et al., 2010). Links between oxidative stress and genotoxicity of ENMs have been demonstrated by several authors (Ali et al., 2020; Raghunathan et al., 2013; Song et al., 2012). The effect did not appear to be influenced by coatings, but the greater DNA damage in hemocytes in CuO core ENM exposures suggests an increased hazard from the uncoated material.

The SOD activity in gill tissues was significantly higher in CuO‐COOH ENM exposures compared with other treatment groups after 48 h of exposure. This was also seen in the gill tissues from 7‐ and 14‐day exposures in Tier 2, suggesting a coating‐dependent effect. Whereas a significant increase in SOD activity was seen in response to CuO‐COOH exposure, CuO core exposure appeared to overwhelm SOD antioxidant systems in gill tissues (lower levels compared with controls at days 7 and 14). In digestive gland tissues the only significant differences in SOD activity levels were measured for CuO‐COOH (day 7) and CuO core exposures (day 21), with no significant effects on TBARS measured, suggesting the gill as the target tissue following CuO ENM exposure.

Tier 3 testing confirmed high Cu accumulation in gill tissues following CuO ENM exposure, as shown in previous studies (Hu et al., 2014). However, although the gill is a primary site of accumulation of Cu^2+^, digestive glands potentially accumulate more CuO ENMs (Chelomin et al., 2017). Thus, the Cu concentrations observed are potentially related to the role of gills in Cu^2+^ processing and may be indicative of the actual concentration of Cu ions that mussels were exposed to following dissolution. Therefore, the accumulation profile likely demonstrates that mussels were exposed to both CuO ENMs and ions to varying degrees according to the coatings present. It is evident that Cu levels increased in all the CuO ENM‐exposed treatment groups after 24 h of depuration, whereas levels remained the same in CuSO_4_ animals, suggesting unique biokinetics for the Cu ion and CuO ENMs. This was also shown by Gomes and colleagues when they compared CuO ENM and CuSO_4_ effects directly (Gomes et al., 2011).

The levels of Cu measured in biodeposits shows that much greater levels of Cu from CuO‐NH_3_^+^–exposed mussels were excreted compared with other treatment groups. The CuO core and PEG‐treated mussels excreted similar levels, whereas CuO‐COOH–exposed mussels excreted less (Supporting Information, Figure S2). Thus, the increased sublethal effects seen in CuO‐COOH–exposed mussels may be related to their slower elimination. In fact, the highest level of uptake in gill tissues was found for CuO‐COOH–exposed mussels. Similarly to the approach used for TiO_2_ ENM bioaccumulation potential comparison (TiO_2_ and coated TiO_2_ ENM hazard and fate assessment using ITS‐ECO approach), if organ‐specific BCF values following uptake (not considering excretion during a depuration phase) were calculated assuming steady state and using the concentration in the tissues divided by the exposure concentration, then gill BCF steady‐state values of 200,000, 300,000, and 50,000 can be derived for CuO‐PEG, ‐COOH, and ‐NH_3_ exposures. These high gill BCF values, calculated to allow comparison, together with the increase in concentrations seen in depurated animals, suggest a potential for bioaccumulation of CuO in mussels that needs further investigation by incorporating longer depuration periods.

Potential bioaccumulation together with the clear cytotoxic, genotoxic, and oxidative stress responses observed suggests that CuO ENMs are a concern for marine compartments.

### Influence of coatings on CuO and TiO_2_ ENM hazard and fate

Coatings had a direct effect on TiO_2_ and CuO ENM size distributions in natural seawater. The PEG and COOH coatings controlled TiO_2_ aggregation with aggregates measuring between 120 and 190 nm, whereas TiO_2_ core suspensions produced aggregates measuring 1300 nm. Even in high salt environments, PEG provides an excellent steric stabilization effect (Guerrini et al., 2018). Carboxylic acids are used as surface modifiers to improve dispersion of ENMs (Sáenz‐Galindo et al., 2018), and thus a stabilizing effect is expected. The amine‐terminated TiO_2_ ENMs did not prevent aggregate formation in seawater with aggregates 900 nm in size, but under HBSS+ conditions, there was a stabilizing effect with similar sizes for all coated TiO_2_ ENMs measured (120–190 nm).

According to bioaccumulation studies, the TiO_2_ core materials were taken up and accumulated in digestive glands (21 days) to a much greater extent than all coated ENMs. Thus, it is likely that the size of the aggregates (affected by the coating) seemingly influenced uptake. The efficiency with which bivalve mussels clear particles from water has been shown to decrease with decreasing particle sizes (Hull et al., 2011). Although particles of 2–60 μm are selected by mussel labial palps, a significant uptake (actively or passively) of these smaller sized TiO_2_ ENMs was seen, which was distinct for particles of approximately 1000 nm compared with particles of approximately 200 nm. Thus, the stabilizing effect of the coating and its influence on aggregate sizes seemingly had a direct effect on bioaccumulation potential in the present study. The lack of significant accumulation for coated TiO_2_ ENMs together with the high amounts of Ti found in pseudofeces suggests that either these smaller aggregates are not taken up (rejected in pseudofeces) or are eliminated quickly.

Despite low levels of accumulation, effects from TiO_2_‐COOH exposure in the gill and digestive gland tissues were found. This could be associated with the negatively charged (anionic) COOH functional groups' increased interaction with tissues and enzymes, because oxidative stress responses following exposure to other COOH‐coated ENMs have been reported (Pichardo et al., 2012). Increased SOD activity levels in gill and digestive gland tissues of CuO‐COOH exposures were also apparent. Therefore, it appears that a COOH coating potentially increases the biological activity/interaction of TiO_2_ and CuO ENMs.

Coatings also seemingly influenced the association of accumulated TiO_2_ core and TiO_2_‐NH_3_ ENMs with pigments hypothesized to be algae or lipofuscin in the digestive gland. Algae‐TiO_2_ aggregates up to 12–50 μm have been observed under acidic pH conditions (pH 4.2; Lin et al., 2015). This interaction is potentially dependent on surface charge, as shown between COO– groups of soluble extracellular polymeric substances (sEPS) of algae and hydrophobic nanoscale (n)TiO_2_ (Adeleye & Keller, 2016). This sorption of sEPS to nTiO_2_ implies that the biomacromolecules may not be easily desorbed from the ENMs and will influence fate and behavior.

Although Raman analysis was not performed for CuO core and CuO‐NH_3_ coatings, the high levels of NH_3_ measured in biodeposits suggest high rejection or elimination of CuO‐NH_3_ ENMs, which was not found in the case of TiO_2_‐NH_3_. Relationships between size and uptake were more difficult to identify for CuO ENMs because similar aggregate sizes were measured in seawater suspensions. However, in the HBSS+ medium used for exposure in hemocytes, CuO‐PEG ENMs were present in the smallest aggregate sizes (310 nm), followed by COOH (987 nm), NH_3_ (1038 nm), and CuO core (1139 nm). Interestingly, the EC50 values also followed this order, suggesting that cytotoxicity was size dependent and greater for smaller aggregate sizes.

Direct surface coating effects on accumulation dynamics and acute toxicity have also been found following exposure of these CuO ENMs to lower trophic level aquatic organisms (e.g., the planktonic crustacean *Daphnia magna*; Gajda‐Meissener et al., 2020).

## CONCLUSIONS

The ITS‐ECO we developed afforded the comparison of hazard potential of the CuO and TiO_2_ ENMs and the various functionalized ENMs (PEG, COOH, and NH_3_) according to levels of cytotoxicity (EC50 values), genotoxicity (Comet assay), and tissue‐specific sublethal early and late oxidative effects (SOD, TBARS) following acute and chronic exposures in *Mytilus* spp. Including fate studies (analysis of uptake in tissues, potential accumulation, and biodeposit analysis) allowed conclusions to be made on possible associations between different levels of uptake/accumulation and biological effects and also the potential for mussels to bioconcentrate such ENMs through excretion in biodeposits. This was clearly shown in the present study for the CuO and TiO_2_ ENMs under investigation and warrants further investigations into potential biomagnification. In addition, information was gained on the influence of functionalizations that could be used in future safe‐by‐design strategies. For example, all coatings significantly reduced the bioaccumulation potential of TiO_2_ ENMs, whereas specific biological interactions with oxidative stress systems (SOD activity levels) were evidenced for COOH‐coated ENMs, in both CuO‐COOH and TiO_2_‐COOH–exposed mussels.

Thus, the ITS‐ECO that we developed serves as an example framework that can be incorporated into specific IATAs for the marine environmental compartment to contribute to the safe and sustainable advancement of nanotechnologies. and indeed such ITS‐ECOs should be developed for other environmental compartments using relevant species.

## Supporting Information

The Supporting Information is available on the Wiley Online Library at https://doi.org/10.1002/etc.5313.

## Author Contribution Statement

**M. Connolly**: Investigation; Methodology; Visualization; Writing—original draft. **S. Little**: Investigation; Methodology; Visualization; Writing—original draft. **M.G.J. Hartl**: Supervision; Writing‐review & editing. **T.F. Fernandes**: Conceptualization; Funding acquisition; Project administration; Supervision; Writing—review & editing.

## Supporting information

This article includes online‐only Supporting Information.

Supporting information.

## Data Availability

All data generated or analyzed during the present study are included in this published article. Data, associated metadata, and calculation tools are also available from the corresponding author (t.fernandes@hw.ac.uk).
