# Supplementary material for: An Integrated Testing Strategy for Ecotoxicity (ITS‐ECO) Assessment in the Marine Environmental Compartment using Mytilus spp.: A Case Study using Pristine and Coated CuO and TiO2 Nanomaterials
Source: Environ Toxicol Chem. 2022 Apr 11;41(6):1390–406. doi: 10.1002/etc.5313 (PMC9321938; doi:10.1002/etc.5313)
Supplement: Supplementary file 1 — Supporting information. [file ETC-41-1390-s001.docx]

Supplementary information

| **NM** | **Surface area^a^**  **(m^2^ g^-1^)**  **Primary size, nm**  **Mean ± SEM [TEM^b^]**  **[TEMc]** | **Hydrodynamic diameter in DI water, nm^d^**  **Pdi^e^** | **Z-potential, mV**  **[DI water] ^f^**  **[Ultrapure water, pH 5]^g^** | **Dissolution rate, μg min^−1^**  **[Ultrapure Water]^h^**  **[Krebs]^i^**  **[NaCl-EBS]^i^** | **Cu content, %**  **[ICP/OES/MS]^j^**  **[TXRF]^k^** |
| --- | --- | --- | --- | --- | --- |
| CuO | 42 ± 2  12.00 ± 0.37  15.9 ± 5.2 | 237 ± 31  0.25 | 27.5 ± 1.8  14.0 ± 1.2 | 0.03  0.02  3.42 | 76.8 ± 5.7  80 |
| CuO−NH_4_^+^ | 6.1 ± 0.5  9.53 ± 0.22  9.2 ± 2.5 | 733 ± 252  0.24 | 25.8 ± 1.3  27.7 ± 0.5 | 0.31  0.20  1.81 | 46.2 ± 4.0  43 ± 2 |
| CuO−COOH | 7.4 ± 0.5  6.45 ± 0.16  6.9 ± 2.2 | 1124 ± 128  0.35 | − 12.0 ± 2.2  - 7.3 ± 0.5 | 1.15  0.30  3.67 | 33.6 ± 3.2  52 ± 2 |
| CuO−PEG | Nd  7.46 ± 0.42  12.1 ± 3.2 | 1244 ± 254  0.35 | − 21.9 ± 3.3  - 16.8 ± 0.4 | 0.87  0.28  2.00 | 11.7 ± 1.0  29 ± 1 |
| TiO_2_ | 98 ± 10 |  |  | nd  < 0.01 Ti*  nd |  |
| TiO_2_- NH_4_^+^ |  |  |  | ^^ |  |
| TiO_2_- COOH |  |  |  | ^^ |  |
| TiO_2_- PEG |  |  |  | ^^ |  |

**Table S1** Physico-chemical properties and characterisation of CuO and TiO_2_ ENMs, adapted from Llves et al. 2019, Kubo et al. 2020 and Vassallo et al. 2018.

a Brunauer–Emmett–Teller (BET) surface area values (mean ± one standard deviation, n = 3) from NANOSOLUTIONS project conducted by A. Besinis

b Based on transmission electron microscopy (TEM) images of CuO ENMs from a 100 mg/L Cu stocks in Milli-Q water where data are mean ± standard error of the mean (S.E.M) with n = 60 measurements (Llves et al. 2019)

c Based on transmission electron microscopy (TEM) images of CuO ENMs from a 200 mg/L Cu stocks suspension in methanol where data are mean ± standard error of the mean (S.E.M) with n = 60 measurements (Kubo et al. 2020)

d Measured by Malvern Zetasizer in 100 mg/l suspension in deionised (DI) water (Kubo et al. 2020)

e Polydispersity index (PDI) (Kubo et al. 2020)

f Measured by Malvern Zetasizer in 100 mg/l suspensions in deionised (DI) water (Kubo et al. 2020)

g Measured by Malvern Zetasizer in 100 mg/l suspensions in ultrapure water defined as Milli-Q water (pH 5), average of five separate measurements (Llves et al. 2019)

hUltrapure water defined as Milli-Q water and dialysis experiments were performed using method of Besinis et al. (2014) with 100 mg/L suspensions at room temperature, pH 5 (Llves et al. 2019)

Krebs defined as Krebs physiological saline and dialysis experiments were performed using method of Besinis et al. (2014) with 100 mg/L suspensions at room temperature, pH 5 Llves et al. 2019) data fitted to a rectangular hyperbola (using SigmaPlot 13), and the maximum initial dissolution rate calculated from the maximum slope (Llves et al 2019)

i NaCl-EBS medium defined as e E-basal salts (EBS) supplemented with 10 mM D-(+)-glucose and dialysis experiments were performed using method of Besinis et al. (2014) with 100 mg/L suspensions at room temperature, pH 6.5 and in triplcate( Vassallo et al. 2018)

j represents the total measured Cu concentration in the original powders of the different test material according to % mass, measured following aqua regia digestion using ICP-OES/ICP-MS (Llves et al. 2019)

k determined using total refection X-ray fuorescence (TXRF)(Kubo et al. 2020)


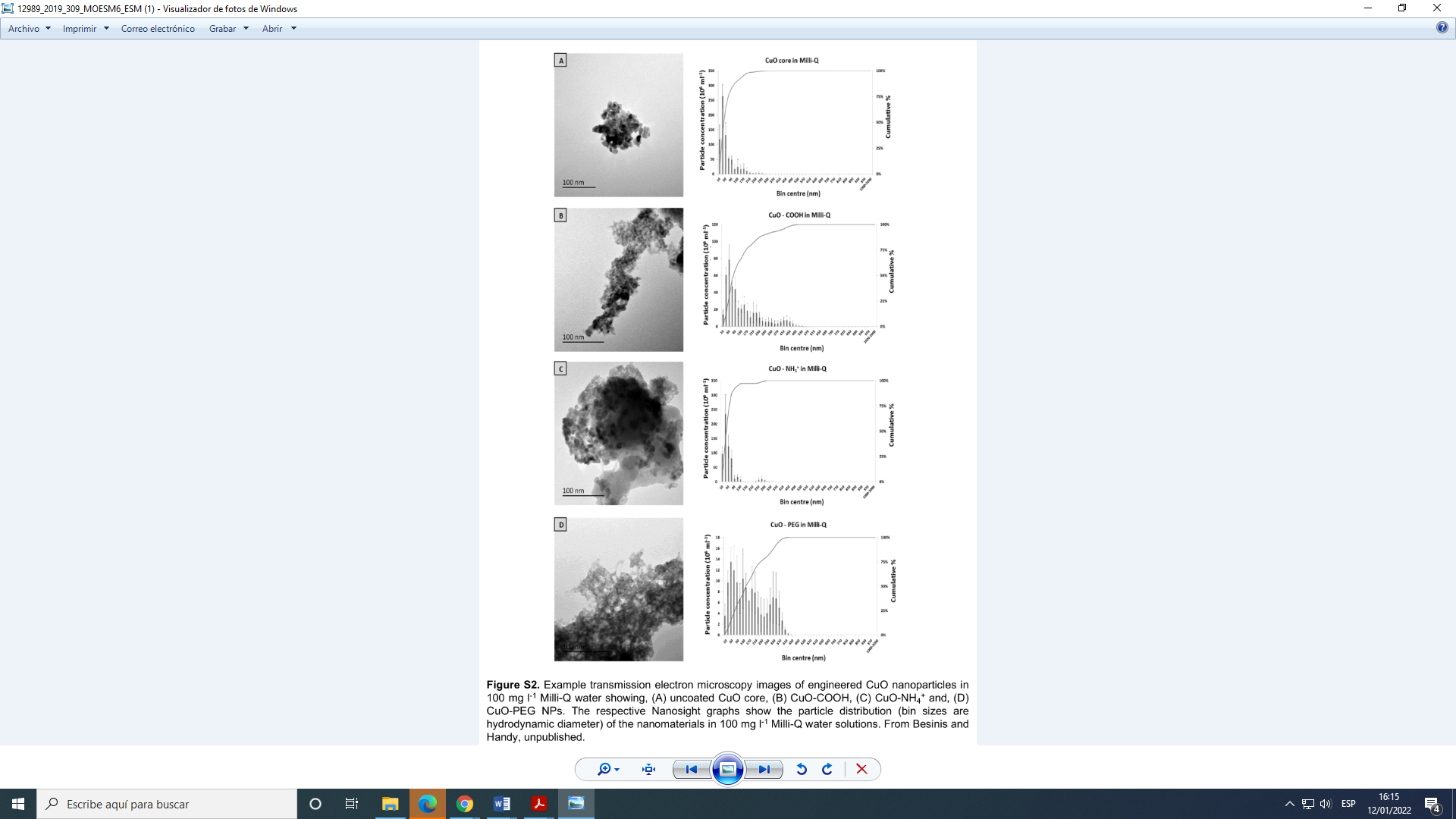

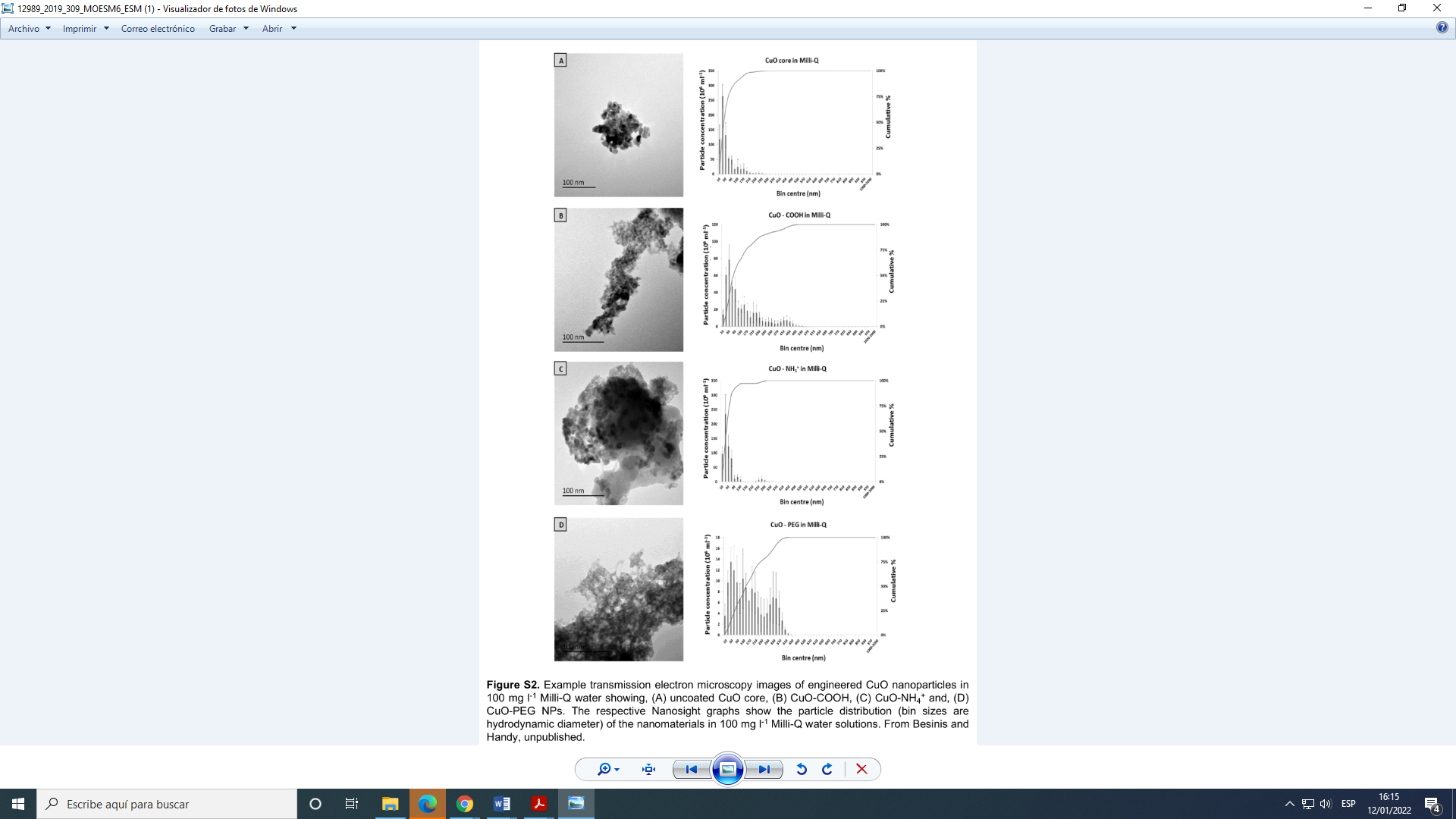


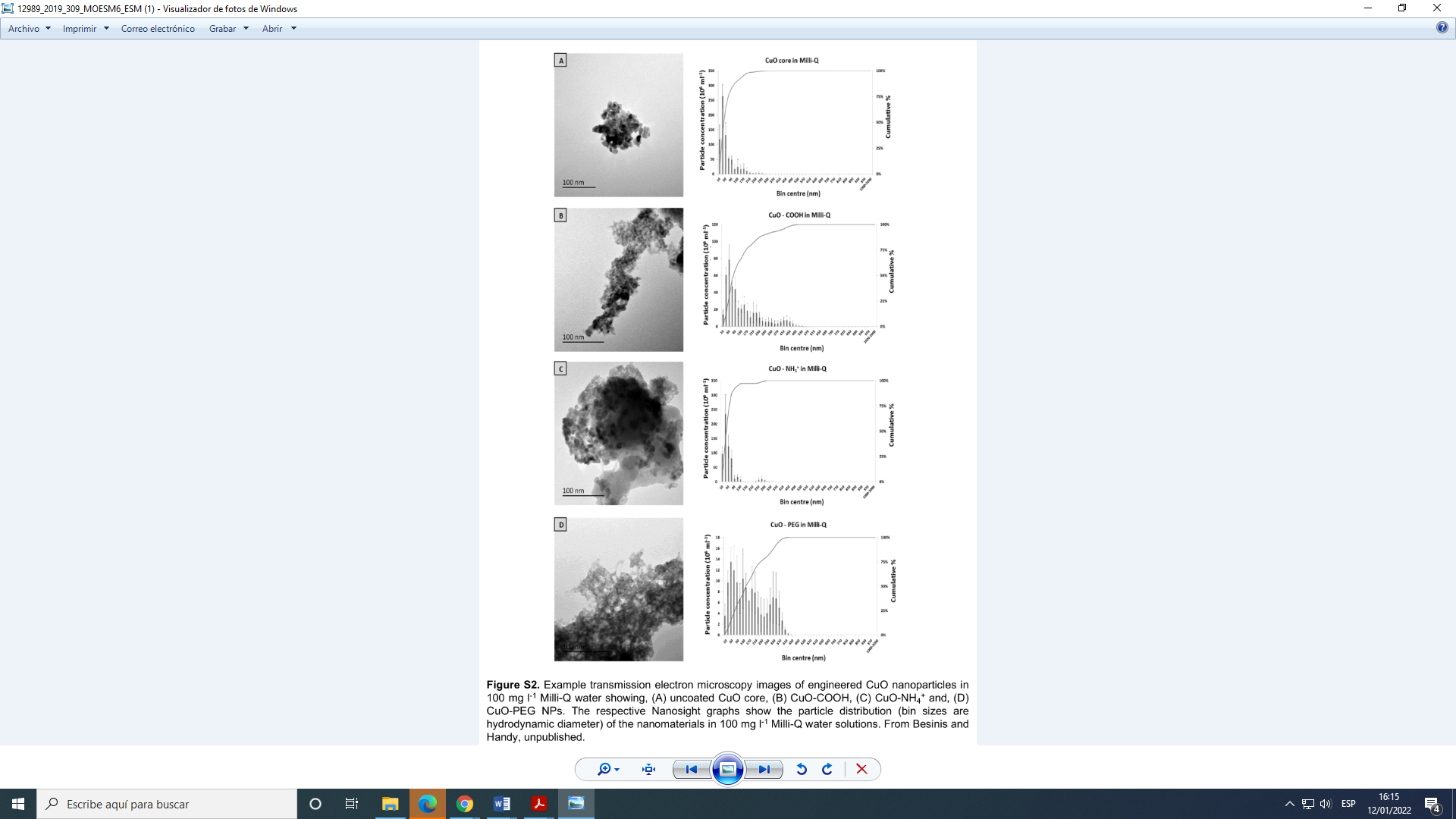

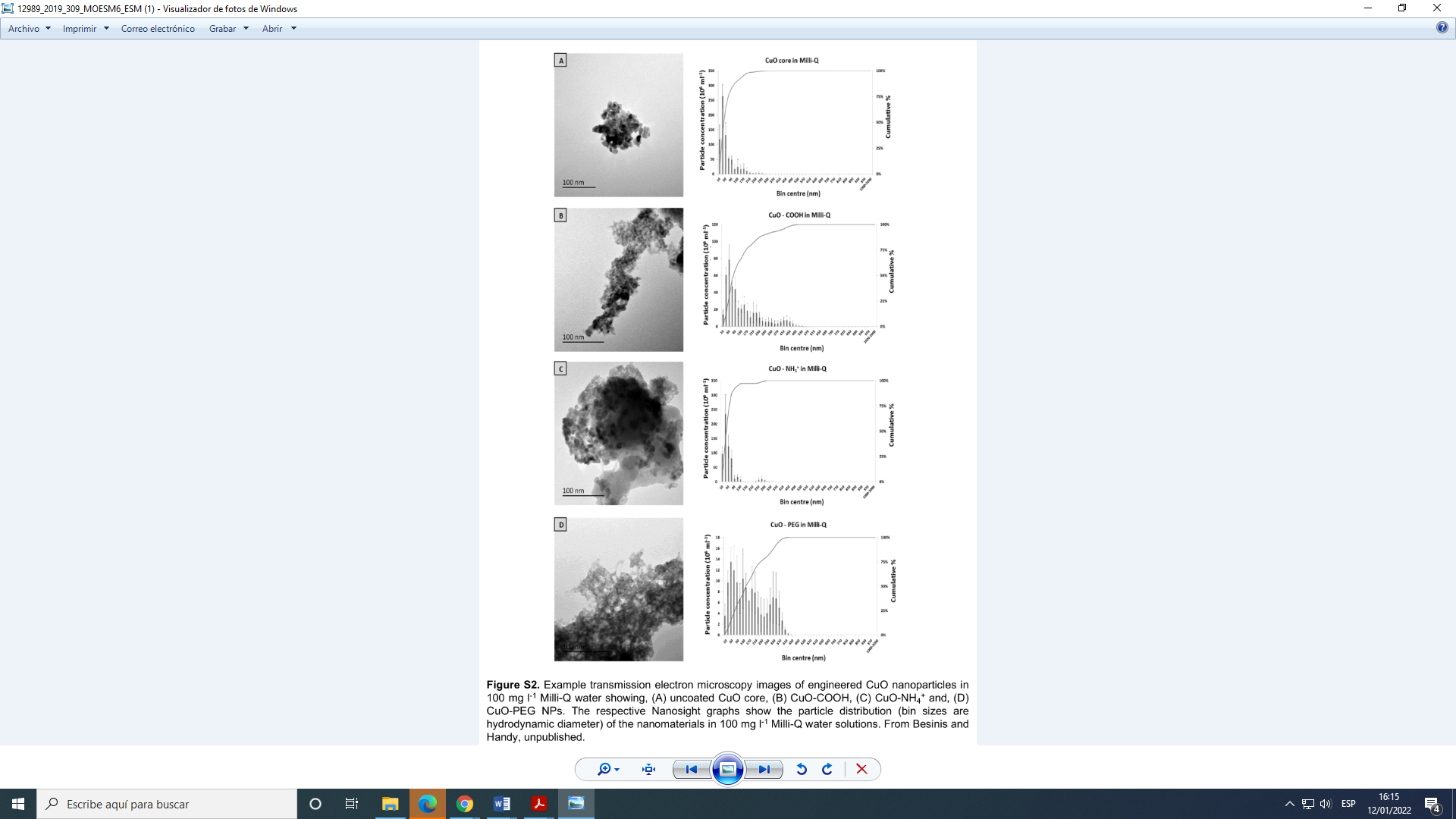


**Figure S1** TEM images of the CuO ENMs dispersed in MiliQ water at 100 mg/L (A) uncoated Core CuO ENMs, (B) CuO-COOH, (C) CuO-NH_3_, (D) CuO-PEG [adapted from Supplementary information in Llves et al. 2019 and originally from Besinis and Handy, unpublished]


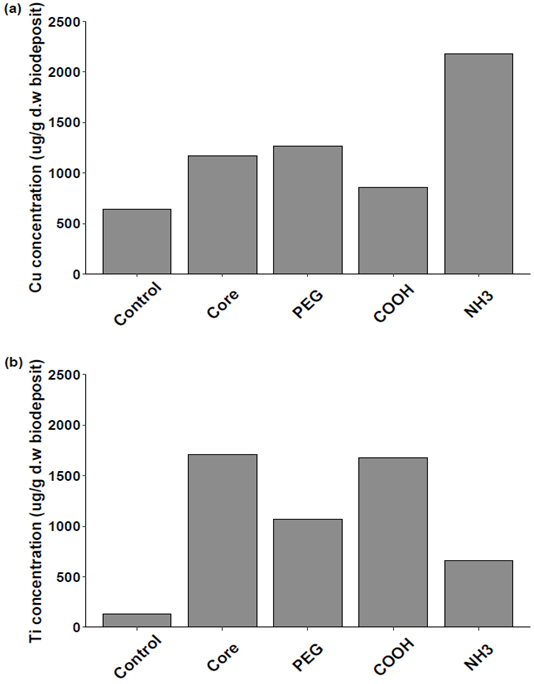


**Figure S2.** Concentrations of Cu (a) and Ti (b) in biodeposits of mussels following 21 day exposure to CuO and TiO_2_ ENMs, respectively. Values represent concentrations measured in a biodeposit sample pooled together from 6 animals and thus mean and error bars cannot be presented or statistical analysis performed.


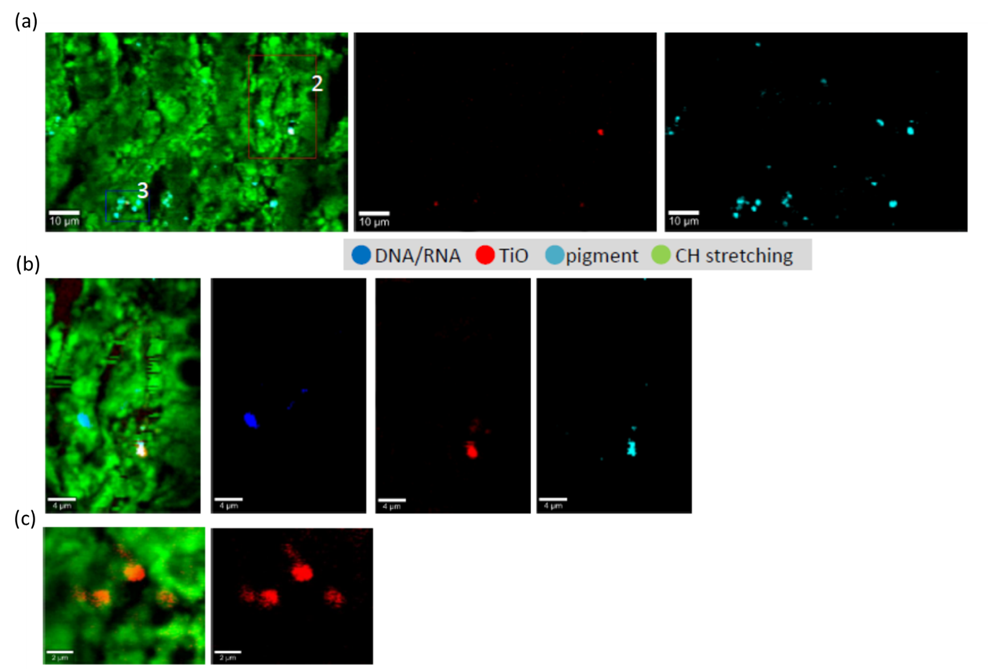


**Figure S3.** Raman mapping of digestive gland tissue of mussels following 21 day exposure to TiO2 NH_3_ NMs. Images on the far left have incorporated all signals including CH stretching from background carbon and hydrogen in tissues (green), DNA/RNA (dark blue), as well as the signal from TiO (red) and a fluorescent pigment (light blue). Individual signals from TiO and the fluorescent pigment by removing the background C-H stretching are also presented. Red and blue rectangles in figure (a) numbered 2 and 3, respectively denote scanned areas with increased magnification and are presented in figures (b) and (c), respectively. Scale bars are 10 µm, 4 µm and 2 µm in figures a, b, and c, respectively.

Comet assay methodology

Microscope slides were evenly frosted with 1% normal gel agarose (NGA) and dried at 40°C. A triple layered agar ‘sandwich’ was then prepared on slides. Briefly, 120 µL 1% NGA was applied to slides, covered with a 22 x 22 mm coverslips and allowed to solidify at 4°C for 20 mins. Coverslips where then removed, 70 µL of low melting point agarose was mixed with 30 µL of either haemolymph or gill samples and applied to the first NGA layer. Coverslips were then replaced and slides refrigerated as previously described. Coverslips were again removed and 120 µL of low melting point agarose was added as a final protective layer and allowed to solidify as before.

Following solidification, coverslips were removed and the cells were lysed in a high salt buffer (2.5 M NaCl, 100 mM EDTA, 1% (v/v) Triton X-100 and 10% (v/v) DMSO, pH 10) for 2 hours in the dark at 4°C. Following lysis, slides were placed into an electrophoresis tank and covered with an alkaline solution (0.3 M NaOH, 1 mM EDTA, pH >12) and left for 30 mins in the dark at 4°C for 30 minutes to allow DNA unwinding. In the same electrophoresis solution, a 25V, 300 mA current was applied for 25 mins, following which slides were neutralised 3 times with Tris buffer (0.4 M Tris-HCl, pH7.4) at 5 min intervals. After washing with distilled water, slides were stained using 5 drops of GelRed® 3X aqueous solution for 5 mins. The slides were again washed with distilled water, following which coverslips were applied to gels. Under an epifluorescence microscope, 50 randomly selected nuclei were analysed using Comet Assay IV software whereby DNA damage was expressed as percentage DNA tail DNA (% of DNA that has migrated from the head).
